# Genetic characterization of *Mycoplasma pneumoniae* isolated in Osaka between 2011 and 2017: Decreased detection rate of macrolide-resistance and increase of *p1* gene type 2 lineage strains

**DOI:** 10.1371/journal.pone.0209938

**Published:** 2019-01-25

**Authors:** Chihiro Katsukawa, Tsuyoshi Kenri, Keigo Shibayama, Kazuo Takahashi

**Affiliations:** 1 Division of Microbiology, Osaka Institute of Public Health, Osaka, Japan; 2 Department of Veterinary Science, Graduate School of Life and Environmental Sciences, Osaka Prefecture University, Osaka, Japan; 3 Department of Bacteriology II, National Institute of Infectious Diseases, Tokyo, Japan; 4 International University of Health and Welfare, Tochigi, Japan; Miami University, UNITED STATES

## Abstract

We characterized 419 *Mycoplasma pneumoniae* isolates collected between 2011 and 2017 in Osaka prefecture of Japan. This analysis revealed high prevalence of macrolide-resistant *M*. *pneumoniae* (MRMP) in Osaka during 2011 and 2014 with annual detection rates of MRMP strains between 71.4% and 81.8%. However, in 2015 and after, the detection rate of MRMP decreased significantly and did not exceed 50%. Genotyping of the *p1* gene of these isolates showed that most of MRMP strains harbored type 1 *p1* gene. In contrast, strains expressing *p1* gene type 2 or its variant were largely macrolide-susceptible *M*. *pneumoniae* (MSMP) strains. There was a strong correlation between *p1* gene genotype and the presence of mutations conferring macrolide resistance in *M*. *pneumoniae* isolated in Osaka. These results indicate that lower incidence of MRMP strains in Osaka from 2015 was associated with the relative increase of *p1* gene type 2 lineage strains. During these experiments, we also isolated three *M*. *pneumoniae* strains that showed irregular typing pattern in the polymerase chain reaction-restriction fragment length polymorphism (PCR-RFLP) analysis of the *p1* gene. Two of these strains harbored new variants of type 2 *p1* gene and were designated as type 2f and 2g. The remaining strain with an irregular typing pattern had a large deletion in the *p1* operon.

## Introduction

*Mycoplasma pneumoniae* is a common bacterial cause of pneumonia and bronchitis in humans [1–3]. Pneumonia caused by this organism accounts for a significant part of community-acquired pneumonia cases worldwide and is particularly common in children and young adults [1,3]. In most cases, symptoms of *M*. *pneumoniae* pneumonia are relatively mild. However, serious cases with various complications that require hospitalization are not uncommon [4]. Macrolide resistance (MR) is a recent global concern for clinical treatment of *M*. *pneumoniae* pneumonia [5,6]. Macrolide-resistant *M*. *pneumoniae* (MRMP) is highly prevalent in Asian countries, including China, Korea, and Japan. Moreover, it is gradually increasing in other areas of the world as well [2,7,8].

**Fig 1:**
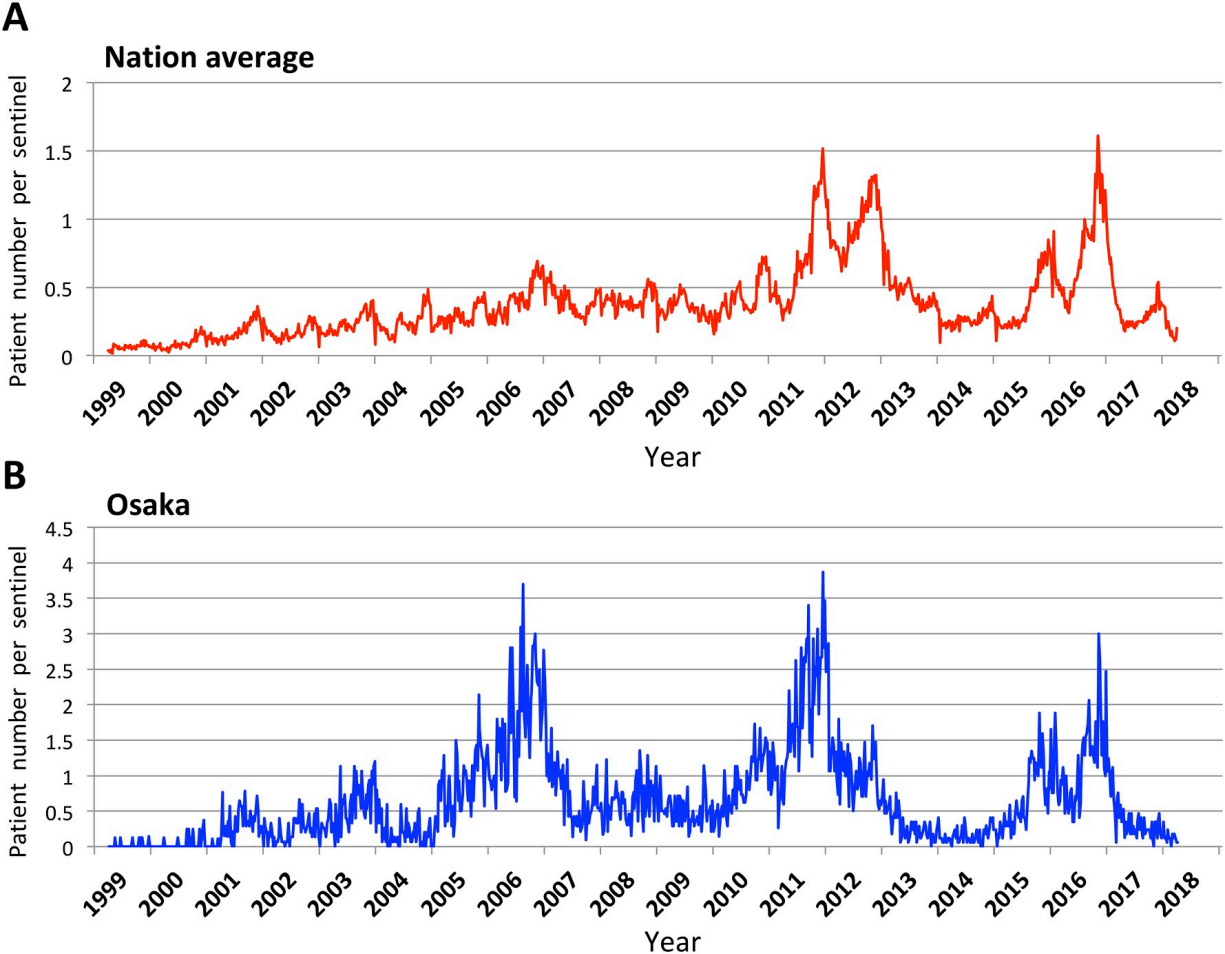
Surveillance of pneumonia cases associated with *M*. *pneumoniae* infection in Japan. Weekly cases of pneumonia caused by *M*. *pneumoniae* in Japan from April 1999 to present. The graphs are based on the data of the National Epidemiological Surveillance of Infectious Diseases (NESID). (A) Nation average is based on the report from about 500 sentinels. (B) The data of Osaka is based on the report from about 7 to 17 sentinels depending on the year. Also see S1 Table for detail. The latest data are available from the website of the National Institute of Infectious Diseases (https://www.niid.go.jp/niid/ja/idwr.html).

Periodical increases in the number of patients with *M*. *pneumoniae* pneumonia in 3–7-year cycles have been reported in many epidemiological studies from various parts of the world [9–14]. In Japan, large epidemics of *M*. *pneumoniae* pneumonia were observed recently in 2011, 2012, 2015, and 2016 by the national epidemiological surveillance of infectious diseases (Fig 1). In the epidemic in 2011 and 2012, high prevalence of MRMP strains among clinical isolates from wide areas of Japan was also reported [15–17].

Currently, the reason for the periodic occurrence of *M*. *pneumoniae* pneumonia epidemics is not fully understood. In addition, it is unclear whether the emergence and wide spreading of MRMP strains affects epidemiological patterns of *M*. *pneumoniae* pneumonia. For better understanding of these aspects of *M*. *pneumoniae* infections, it is important to isolate *M*. *pneumoniae* strains from patients continuously and to examine their genetic properties. In this study, *M*. *pneumoniae* isolates were collected from 419 patients with respiratory infections between 2011 at 2017 in Osaka prefecture, the second largest metropolitan area in Japan (population of Osaka prefecture is approximately 9 million). We report here the results of MR analysis and genotyping of these *M*. *pneumoniae* isolates.

## Materials and methods

### Isolation of *M*. *pneumoniae*

From July 2011 to March 2017, throat swabs were collected from patients suspected of *M*. *pneumoniae* infection in six pediatric clinics and nine pediatrics departments of hospitals in Osaka prefecture. The swabs were collected based on the approvals of the Ethics Committee of the Osaka Institute of Public Health (1602–05 and 1310–02). Written informed consent was obtained from the parents or guardians of all subjects. The swabs were kept in BD Universal Viral Transport System (Nippon Becton Dickinson Inc., Tokyo, Japan) and cryopreserved until further examination. The specimens were inoculated into both diphasic mycoplasma medium and PPLO broth [18] and cultured at 37 °C for 2 months. Culture-positive specimens during the culture period were stored, and identification of *M*. *pneumoniae* was performed by PCR [19].

### Detection of MR mutations in the 23S rRNA gene

MR of *M*. *pneumoniae* isolates was determined by sequencing of the domain V region of the 23S rRNA gene. PCR primers Myco23S-F (5′-TCTCGGCTATAGACTCGGTGA-3′, positions 1998–2018 of the 23S rRNA gene) and Myco23S-R (5′-TAAGAGGTGTCCTCGCTTCG-3′, positions 2673–2692) were designed and used to amplify the region that contains the known MR mutation sites (in mycoplasma numbering, positions 2063, 2064, 2067, and 2617 of 23S rRNA gene). The amplified PCR products were sequenced by using the same primers on an ABI 3130 genetic analyzer (ThermoFisher Scientific, Waltham, MA, USA) to detect the mutations.

### *P1* gene typing of *M*. *pneumoniae* isolates

Isolate *p1* gene (MPN141) types were determined by using the polymerase chain reaction-restriction fragment length polymorphism (PCR-RFLP) typing method [20–22]. Briefly, genomic DNA of the isolates was extracted from the PPLO culture medium using a QIAamp DNA Mini Kit (QIAGEN, Tokyo, Japan). Two pairs of primers (ADH1-ADH2 and ADH3-ADH4) were used to amplify the polymorphic regions of the *p1* gene (containing RepMP4 or RepMP2/3 regions) by PCR from genomic DNA. PCR products were digested with *Hae*III restriction endonuclease and electrophoresed on a 2% agarose gel. After gel staining, RFLP patterns of the PCR products were compared to determine *p1* types.

### Sequencing analysis of the *p1* operon region of new variant strains

The region containing the entire *p1* gene of the new variant strains M282 and K708 was amplified by PCR from genomic DNA using primers P1-200F (5′-GGCTGTTCTTTATAGAAGAG-3′) and P1+100R (5′-TGGTCTTGGAGGAGGTAGGT-3′). The *orf6* gene (MPN142) of strains M282 and K708 was also amplified by PCR using primers ORF6-F (5′-GCGCCAAAACGCTTGAAACA-3′) and OP-R1 (5′-TTGCACTAGGAAGGTAATGT-3′). The *p1* operon region of strain M241 was amplified by using forward primers OP-F1 (5′-TACTACTTACAACTCTTTGT-3′) or ADH3 (5′-CGAGTTTGCTGCTAACGAGT-3′) and the reverse primer 23e-R1 (5′-AAGAGGTGAAGCCTCGCTAA-3′). Amplified DNA fragments were sequenced by the primer-walking strategy using an ABI 3130xl genetic analyzer (ThermoFisher Scientific). The primer sequences used for the primer-walking of the *p1* operon region are listed in S2 Table.

### Statistical analysis

Data were analyzed by the Fisher’s exact test with a significance level of α = 0.05 (*P* < 0.05) by using R software version 3.2.3.

### Nucleotide sequence accession numbers

Nucleotide sequences of *p1* genes of type 2f strain M282 and type 2g strain K708 were deposited into DDBJ/ENA/GenBank databases under the accession numbers LC311244 and LC385984, respectively. The sequences of the *orf6* gene of strains M282 and K708 were deposited under the accession numbers LC390170 and LC420352, respectively. The sequence of the *p1* operon of strain M241 was also deposited under the accession number LC390171.

## Results

### Variable annual detection rate of MRMP strains in Osaka prefecture

In this study, a total of 419 *M*. *pneumoniae* strains were isolated from patients with suspected *M*. *pneumoniae* infection in Osaka prefecture between 2011 and 2017. Table 1 shows the numbers of isolated *M*. *pneumoniae* strains per annum. Numbers of isolates differed from year to year, and their isolation frequency correlated well with the annual prevalence of *M*. *pneumoniae* pneumonia in Osaka prefecture (Fig 1).

**Table 1:** Number of *M*. *pneumoniae* isolates collected in this study and macrolide resistance rate.

Year	Number of isolates	Number of MR isolates	MR rate (%)
2011	64	49	76.6
2012	11	9	81.8
2013	7	5	71.4
2014	15	11	73.3
2015	157	65	41.4
2016	157	67	42.7
2017	8	4	50.0
Total	419	210	50.1

All MR isolates harbored A2063G mutation except for one isolate that had A2063T mutation. The isolate that harbored A2063T was obtained in 2013. MR: macrolide-resistance. Also see S3 Table.

To determine the prevalence of MR in these isolates, we analyzed the domain V region of the 23S rRNA gene in all isolates by PCR and DNA sequencing. MR mutations were found in 210 out of 419 isolates (50.1%). The fraction of MRMP strains was higher than 70% between 2011 and 2014 (71.4–81.8%), whereas in 2015 and after, it decreased significantly to 41.4–50% (*P* < 0.01, Fisher’s exact test) (Table 1). All of MRMP isolates but one (209/210, 99.5%) harbored the A2063G transition mutation, the most frequent MR mutation reported to date [7]. One strain isolated in 2013 had A2063T transversion (Table 1).

We also compared the fractions of isolates with MR in clinics (primary medical institutions) with that in hospitals (higher order medical institutions) in 2015 and 2016 to establish if MR incidence depended on the scale of the medical institutions (Table 2). In 2015, the fractions of MR isolates were significantly higher in hospitals (59/110, 53.6%) than in clinics (6/47, 12.8%; *P* < 0.01, Fisher’s exact test). Similar tendency was also observed in 2016. The fraction of MR isolates in 2016 was 45.9% (62/135) in hospitals and 22.7% (5/22) in clinics (*P* = 0.061, Fisher’s exact test). Thus, the incidence of MR in isolates from hospitals was higher than that in isolates from clinics.

**Table 2:** Differences in the rate of macrolide resistance of *M*. *pneumoniae* isolates between clinics and hospitals.

Year	Origin of isolates	Number of isolates	Number of MR isolates	MR Rate (%)
2015	Clinic	47	6	12.8
	Hospital	110	59	53.6
2016	Clinic	22	5	22.7
	Hospital	135	62	45.9

MR: macrolide resistance

### Relationship between *p1* gene type and resistance of *M*. *pneumoniae* isolates to macrolides

By using the PCR-RFLP method [22], we carried out *p1* gene typing of 419 isolates. As a result of this typing analysis, 223 isolates were classified as type 1 (53.2%), 102 isolates were type 2 (24.3%), 6 were type 2a (1.4%), 1 was type 2b (0.2%), 84 were type 2c (20%), and 3 were non-typable (0.7%) (Table 3). The *p1* gene type 1 isolates were highly prevalent between 2011 and 2014 (71.4–90.9%), however after this period, type 2 and type 2c isolates increased significantly (*P* < 0.01, Fisher’s exact test). Therefore, the relative isolation rate of type 1 *M*. *pneumoniae* strains decreased to 43.3–62.5% between 2015 and 2017 (Table 3).

**Table 3:** Annual distribution of *p1* gene typing results in *M*. *pneumoniae*.

Year	*p1 gene* type						Total
	1	2	2a	2b	2c	Non-typable*	
	(%)	(%)	(%)	(%)	(%)	(%)	
2011	53	0	6	1	4	0	64
	(82.8)		(9.4)	(1.6)	(6.3)		
2012	10	0	0	0	1	0	11
	(90.9)				(9.1)		
2013	5	0	0	0	2	0	7
	(71.4)				(28.6)		
2014	11	0	0	0	4	0	15
	(73.3)				(26.7)		
2015	68	47	0	0	42	0	157
	(43.3)	(29.9)			(26.8)		
2016	71	55	0	0	28	3	157
	(45.2)	(35)			(17.8)	(1.9)	
2017	5	0	0	0	3	0	8
	(62.5)				(37.5)		
Total	223	102	6	1	84	3	
%	(53.2)	(24.3)	(1.4)	(0.2)	(20)	(0.7)	419

Annual and total percentages of each *p1* types are shown in parentheses.

* Strains M241, M282 (type 2f), and K708 (Type 2g). See text.

Type 2b is also referred as type 2V in other studies [26,49].

We then examined whether *p1* typing results correlated with the presence of MR mutations in isolates and found that the presence of MR mutations in isolates was highly dependent on *p1* gene type. Most of type 1 isolates harbored MR mutations (204/223 isolates, 91.5%). In contrast, only one type 2 (1/102, 1%) and five type 2c (5/84, 6%) isolates harbored A2063G mutation (Table 4). The total isolates with MR mutation in type 2 lineage (2, 2a, 2b, and 2c) was 3.1% (6/193). Thus, the presence of MR mutations was strongly determined by the *p1* type (between type 1 and 2 lineages) (*P* < 0.01, Fisher’s exact test).

**Table 4:** Distribution of MR mutations in *M*. *pneumoniae* isolates of different *p1* types.

Macrolide-resistant mutations	*p1* type						Total
	1	2	2a	2b	2c	Non-typable*	
	(%)	(%)	(%)	(%)	(%)	(%)	
None	19	101	6	1	79	3	209
	(8.5)	(99)	(100)	(100)	(94)	(100)	
A2063G	203	1	0	0	5	0	209
	(91)	(1)			(6)		
A2063T	1	0	0	0	0	0	1
	(0.5)						
Total	223	102	6	1	84	3	419
Total MR (%)	(91.5)	(1)	(0)	(0)	(6)	(0)	(50.1)

MR and MS percentages of each *p1* type isolates are shown in parentheses.

*Strains M241, M282 (type 2f), and K708 (Type 2g). See text.

Type 2b is also referred as type 2V in other studies [26,49].

**Fig 2:**
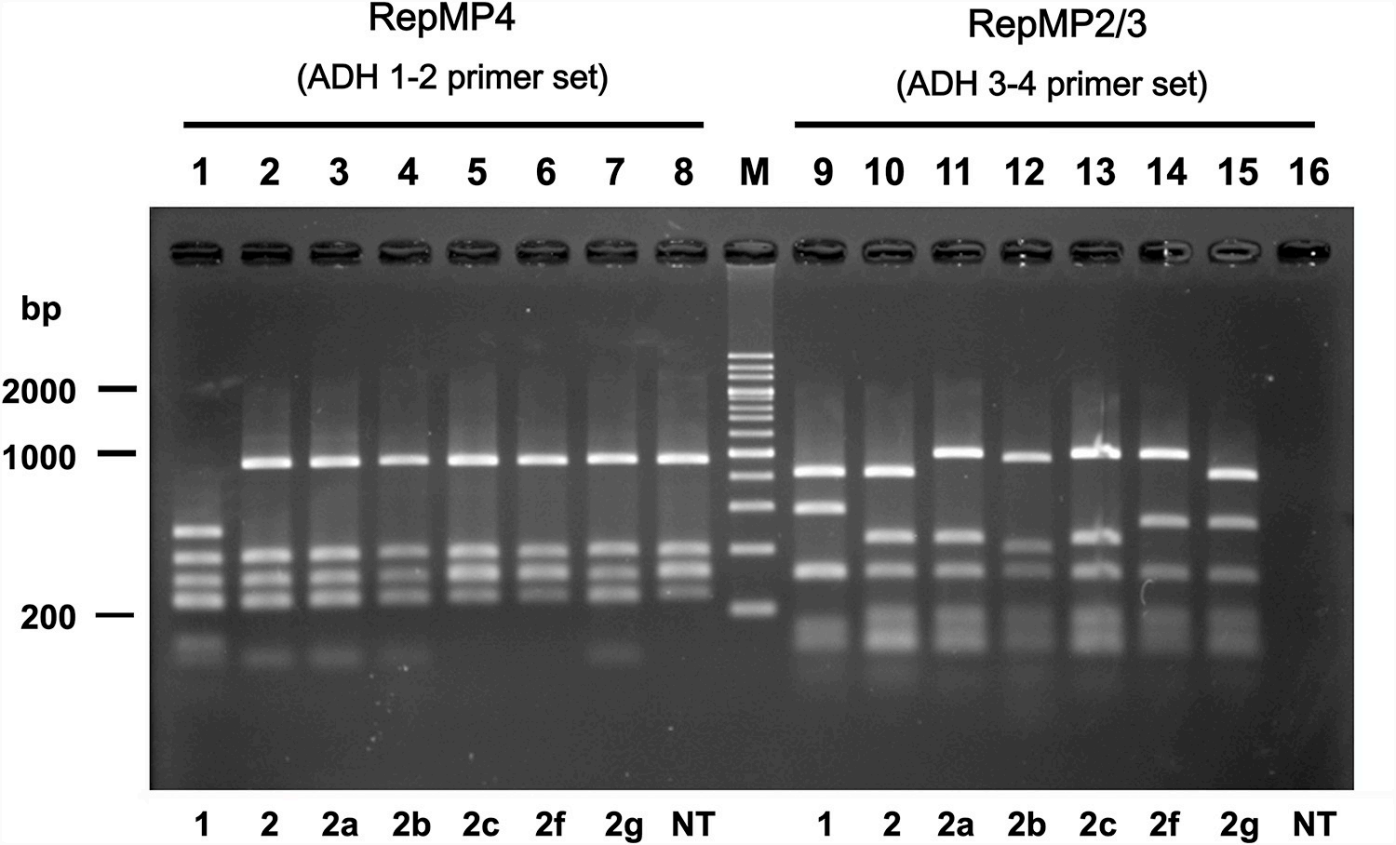
PCR-RFLP typing of the *p1* gene. *M*. *pneumoniae* isolates from 419 patients in this study were classified into eight *p1* gene types by PCR-RFLP analysis. The RFLP patterns of the RepMP4 region (lanes 1 to 8) and the RepMP2/3 region (lanes 9 to 16) that represent eight *p1* gene types are shown. *p1* gene types corresponding to RFLP patterns are indicated below the gel image. NT indicates non-typable strain M241 (see text). Lane M contains size markers (200 bp ladder). Type 2b is also referred as type 2V in other reports [26,49]. The original electrophoresis image is shown in S3 Fig).

Three non-typable *p1* isolates were collected in 2016 and designated as strains M241, M282, and K708 (Tables 3 and 4). In the RepMP4 region analysis, these strains exhibited known PCR-RFLP patterns. The patterns of strains M282 and M241 were identical to that of type 2c strains (Fig 2, lanes 6 and 8). Furthermore, the RepMP4 pattern of strain K708 was identical to that of type 2, 2a, or 2b strains (Fig 2, lane 7). RepMP2/3 region patterns of these strains were however different from those of known strain types (Fig 2, lanes 14–16). RepMP2/3 patterns of strains M282 and K708 were novel (Fig 2, lanes 14 and 15). The RepMP2/3 region of strain M241 could not be analyzed due to the lack of PCR amplification when ADH3 and ADH4 primers were used (Fig 2, lane 16).

### Characterization of the *p1* operon region of M241, M282, and K708 strains

**Fig 3:**
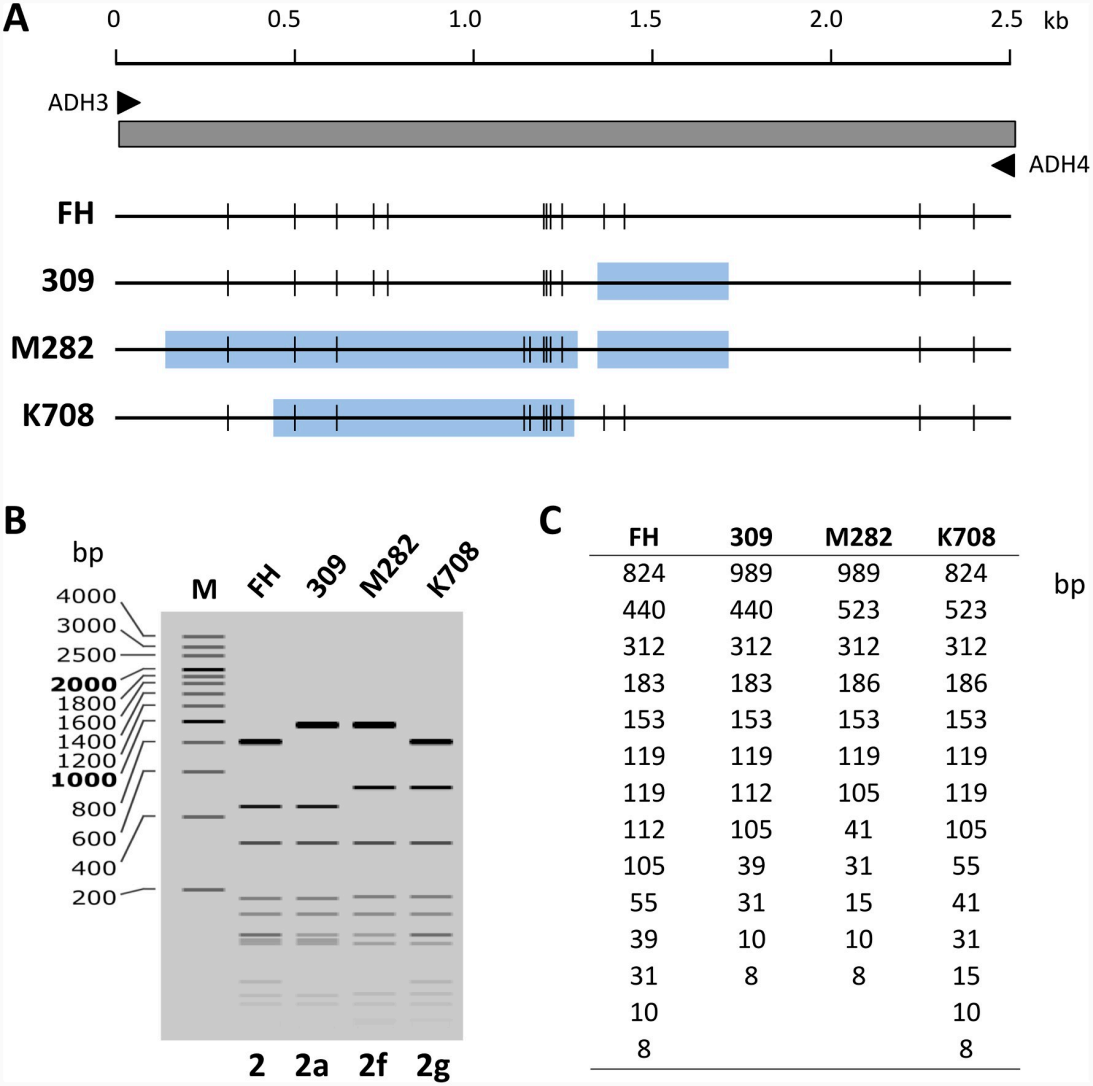
Novel *p1* gene sequence variations in strains M282 and K708. (A) Schematic diagram of the PCR amplicons obtained using the primers ADH3 and ADH4, containing RepMP2/3 region of the *p1* gene. Predicted *Hae*III sites within the amplicons from strains FH (type 2), 309 (type 2a), M282 (type 2f), and K708 (type 2g) are shown. Light blue boxes indicate the regions of sequence variations compared to that of the type strain FH. (B) Simulated pattern of 2% agarose electrophoresis of *Hae*III-digested amplicon fragments predicted by using the *p1* gene sequences (GenBank accession nos. CP010546, AP012303, LC390170 and LC385984). The pattern was calculated by using SnapGene software version 4.2.3 (Snapgene.com). (C) Theoretical size of *Hae*III-digested amplicon fragments.

To establish the reason for the irregular RFLP patterns of strains M241, M282, and K708 (Fig 2), we analyzed the *p1* gene region of these strains by sequencing. The entire *p1* gene regions of strains M282 and K708 were amplified from the genomic DNA by PCR and sequenced. These analyses identified novel sequence variations in the RepMP2/3 region of strains M282 and K708. The theoretical *Hae*III digestion patterns of ADH3-4 amplicon were in good agreement with experimental data (Fig 2, lanes 14 and 15; Fig 3). In the *p1* gene region of strain K708, a minor variation was also identified in the RepMP4 region (S1 Fig). This minor variation in the RepMP4 region did not affect the *Hae*III site and RFLP pattern. The *p1* genes of M282 and K708 strains are thought to have originated from type 2c and type 2 *p1* genes, respectively. However, novel variation sequences of these genes were partially similar to the type 1 *p1* gene sequence (S1 Fig). We therefore designated the *p1* genes of strains M282 and K708 as type 2f and 2g, respectively (GenBank accession nos. LC311244 and LC385984). These variations were presumably generated by gene conversion-like DNA recombination between RepMP2/3 sequences within and outside of the *p1* operon [23,24] (S1 Fig).

The recently obtained *M*. *pneumoniae* whole genome sequence datasets suggest that *p1* gene type 2b and 2c strains have common sequence variations in the *orf6* gene (MPN142), which differentiate these strains from other type 2 strains [25,26]. Therefore, we have sequenced the *orf6* region of strains M282 and K708 and revealed that the type 2f strain M282 had a novel, 90-bp long sequence variation at the 5′ end of the *orf6* gene (GenBank accession no. LC390170) (S1 Fig). Furthermore, the *orf6* sequence of the type 2f strain K708 (GenBank accession no. LC420352) was identical to that of the type 2 strain FH (GenBank accession no. CP017327) (S1 Fig).

The *p1* gene of strain M241 could not be amplified with PCR primers used for the analysis of M282 and K708 *p1* genes. Because a large sequence change was expected at the 3′ end of M241 *p1* gene, we designed several PCR primers that correspond to downstream of the *p1* operon. Using one of these reverse primers, designated as 23e-R1, which was specific to the site about 2.5 kb downstream of the *p1* operon (downstream of the *orf6* gene), we achieved amplification of M241 *p1* gene region. The PCR product size was about 6.8 kb shorter than expected (S2 Fig). Sequencing of the PCR product revealed that a 6.8-kb deletion occurred in the *p1* operon of M241 strain due to DNA recombination between the RepMP2/3 region in the *p1* gene and another RepMP2/3 located downstream of the *p1* operon (GenBank accession no. LC390171). This deletion caused a loss of the ADH4 primer binding site and entire *orf6* gene (S2 Fig). As expected from these results (truncation of the 3′ end of the *p1* gene and loss of the *orf6* gene), strain M241 had no cytadherence activity in the conventional hemadsorption assay [27,28].

## Discussion

**Fig 4:**
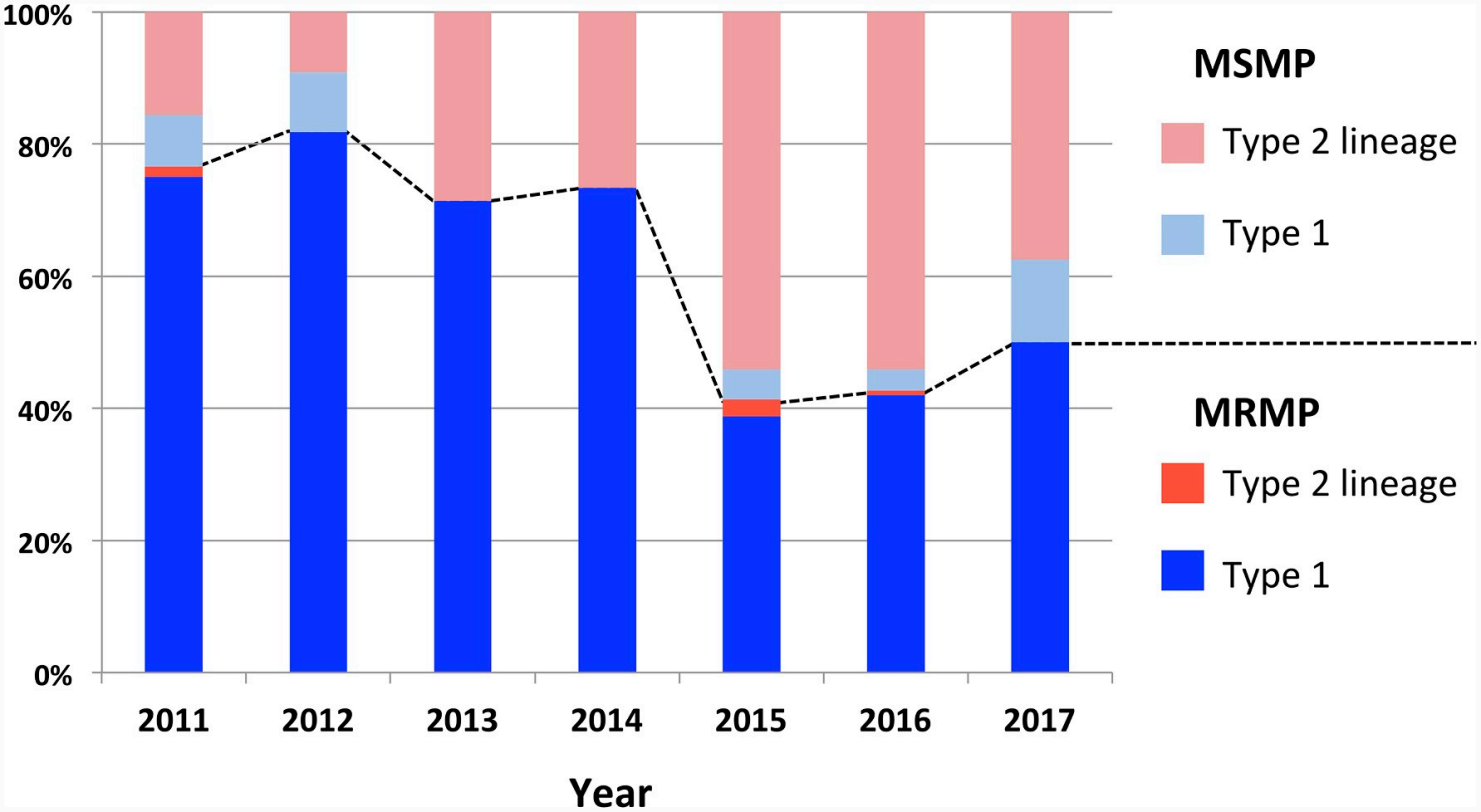
Annual rates of isolation of macrolide-resistant *M*. *pneumoniae* (MRMP) and macrolide-susceptible *M*. *pneumoniae* (MSMP) strains in Osaka in 2011–2017. The fractions of *p1* gene type 1 and type 2 lineage MRMP isolates are shown in blue and red, respectively. The fractions of *p1* gene type 1 and type 2 lineage MSMP isolates are shown in light blue and pink, respectively. Dotted line indicates the boundary between MRMP and MSMP strains. Also see S4 Table.

In this study, we collected 419 *M*. *pneumoniae* isolates in Osaka prefecture between 2011 and 2017, established their *p1* gene type, and identified MR-conferring mutations. Several previous studies reported that the dominant *p1* type of *M*. *pneumoniae* clinical isolates in Japan was type 1 since 2003 [1,29]. This was also true in the large epidemic period in 2011 and 2012 [15,30]. Our study in Osaka was consistent with these previous reports, showing high prevalence of type 1 isolate between 2011 and 2014 (Table 2). However, after that period, type 2 lineage (type 2 and 2c) isolates increased rapidly and comprised more than half of all isolates in 2015 and 2016 (Table 2). Interestingly, in contrast to type 1 isolates that are largely MRMP strains, most of type 2 lineage isolates were MSMP strains (Table 4 and Fig 4). Therefore, the total ratio of MR strains among *M*. *pneumoniae* isolates in Osaka decreased in 2015 and thereafter (Table 1 and Fig 4). Low prevalence of MR in *p1* type 2 strains has already been reported in *M*. *pneumoniae* isolates in Yamagata prefecture of northern Japan between 2004 and 2014 [16,30]. In addition, a recent epidemiological study of *M*. *pneumoniae* pneumonia in Japan reported nationwide decrease of MRMP strains after 2013, although *p1* typing was not performed [31]. In that nationwide report, publication of treatment guidelines for *M*. *pneumoniae* pneumonia is mentioned as one of the major factors for the decrease of MRMP strains in Japan after 2013. With these guidelines published in 2011, clinicians may have started paying more attention to the appropriate use of macrolides to prevent the spread of MRMP. This may partly explain the decrease of MRMP strains in Japan after 2013. However, as shown in our study in Osaka, we consider that the increase in MSMP strains with *p1* gene type 2 lineages was another major factor for the decrease of MR rate in Japan.

The real mechanism underlying the correlation between MR and *p1* gene type revealed in this study is unknown, however the probable reasons may be as follows. Type 2 *M*. *pneumoniae* was frequently isolated in Japan in 1990s [1,29]. Therefore, in early studies of MR of *M*. *pneumoniae* in Japan, no correlation between MR and *p1* gene type was noted [32,33]. However, after 2003, type 1 *M*. *pneumoniae* became dominant, and this continued for about 10 years in Japan [1,15,16,29,30]. Most of *M*. *pneumoniae* pneumonia cases in that period might have been caused by type 1 *M*. *pneumoniae*. In this situation, chemotherapy using macrolides might select resistant strains from type 1 *M*. *pneumoniae* rather than type 2 lineage. Then, type 1 MRMP strains spread widely in the community until the epidemic period in 2011 and 2012. However, after then, the occurrence of *M*. *pneumoniae* type 2 lineage increased, probably because of the type shift phenomenon of the pathogen as a result of interactions between antigenicity of pathogens and immunological status of human population [1,29,34]. At present, type 2 lineage strains isolated from patients are still considered as being macrolide-sensitive. To assess these hypotheses, it is particularly important to continue surveillance and carry out genetic characterization of *M*. *pneumoniae* isolates to establish whether the number of MRMP isolates of *p1* type 2 lineage increases after this point.

Correlation between MR and *p1* type of *M*. *pneumoniae* has also been reported in studies of recent clinical isolates in China and Korea [35–39]. Probably, the clinical usage of macrolides and dominant *p1* gene type trend in these countries are similar to those in Japan. Geographic location may also affect distribution of similar *M*. *pneumoniae* strains. However, MR and *p1* type did not correlate closely in the studies performed in the United States and other countries [40–42]. In the United States, the MR rate did not change over time despite fluctuations in dominant *p1* type [42,43]. The lack of correlation may be attributed to the generally low MR rate in these countries at the time of observation (under 30%), similar to the situation in Japan around year 2000 [32,33]. The preservation of the low MR rate of *M*. *pneumoniae* in these countries may be related to the frequency of macrolide usage in clinical treatments in these countries. The selection of MRMPs with specific *p1* type has not yet occurred in these countries.

In Japan, macrolides are recommended as first-line drugs for the treatment of *M*. *pneumoniae* pneumonia [1]. If macrolides are not effective, tetracyclines and fluoroquinolones are recommended as second-line drugs. However, the use of tetracyclines in children younger than 8 years old presents a significant risk because of adverse effects. There is also an ongoing debate regarding the therapeutic effects of fluoroquinolones (tosufloxacin) used for the treatment of *M*. *pneumoniae* pneumonia in children [17,44]. Therefore, providing updated information on the fraction of MRMP isolates is important for effective treatments of diseases by clinicians. However, the fraction of MRMP strains inferred by the epidemiological studies may be different from the actual prevalence of MR in the community, depending on the study design. In other words, MR rate may be higher in large hospitals than in clinics because *M*. *pneumoniae* pneumonia patients who do not recover after the treatment with macrolides and exhibit severe symptoms tend to transfer from clinics to larger hospitals. In these serious cases, pathology is usually caused by MRMP strains. Therefore, we compared the fractions of MR isolates in clinics and in hospitals in 2015 and 2016. As expected, the MR rate in hospitals was higher than that in clinics (Table 2). However, the MR rate in clinics rather than in hospitals may better reflect actual prevalence of MRMP strains in the community.

In this study, we have identified new type 2 variant *p1* genes designated as type 2f and 2g (GenBank accession nos. LC311244 and LC385984). These type 2 variants partially encompassed type 1 *p1* gene sequences derived from the RepMP4 sequence outside the *p1* operon (S1 Fig). New *p1* variant strains may appear spontaneously at a low frequency in the population of *M*. *pneumoniae* due to shuffling of RepMP sequences mediated by gene conversion-like DNA recombination [23,24,45]. However, the fate of new *p1* strains, i.e., whether they become a major subtype or disappear, may depend on natural selection, survival of the fittest, or chance. It is empirically known that *p1* types of isolates do not change easily during repeated passages in laboratory. The stability of *p1* type may be related to the low level of DNA recombination in this bacterium [46,47]. Therefore, we believe that type 2f and 2g strains isolated in this study were the cause of pneumonia and were not created during culture isolation, although we did not confirm this by direct *p1* typing of clinical specimens. Furthermore, another isolate, M241, displayed no cytadherence activity due to a large deletion in the *p1* operon (S2 Fig). Cytadherence is an essential factor for *M*. *pneumoniae* pathogenesis, therefore, there is a possibility that M241 was not involved in the disease onset and was selected or emerged during culture isolation. Strain S355 isolated in China (GenBank accession no. CP013829) also had a large deletion similar to that in M241 in the *p1* operon probably due to DNA recombination between RepMP4 sequences, although the phenotype of that strain has not been described [48]. Spontaneous loss of functional *p1* operon by recombination between RepMP sequences may occur more frequently than generation of new *p1* variants in the population of *M*. *pneumoniae* (S2 Fig).

In this study, we have characterized 419 *M*. *pneumoniae* isolates collected in Osaka between 2011 and 2017. This analysis revealed a correlation between the decrease of MRMP strains and increase of *p1* gene type 2 lineage *M*. *pneumoniae* in Japan in recent years. The limitation of this study was the highly variable number of strains isolated in different years, which ultimately depended on the annual number of pneumonia patients (Fig 1 and Table 1). This made the comparison of MR rate and *p1* type data between different years complicated. However, information obtained in our present analyses may be useful for future epidemiological studies. Continuous efforts for the collection of *M*. *pneumoniae* isolates and genotyping analysis are important for better understanding of epidemiology of *M*. *pneumoniae* infections.

## Supporting information

S1 Fig Comparison and classification of *p1* and *orf6* gene subtypes in *M*. *pneumoniae*. (A) Schematic illustration of the *p1* operon region and comparison of 11 *p1* subtypes. Three light blue arrows indicate *orf4* (MPN140), *p1* (MPN141), and *orf6* (MPN142) genes of the *p1* operon. The approximate size of the operon is shown above in base pairs (bp). The positions correspond to P40 and P90 proteins are shown in the *orf6* gene. Pink, green, and blue rectangles below the *p1* and *orf6* genes indicate approximate positions of RepMP repetitive sequence regions (RepMP4-c, RepMP2/3-d, and RepMP5-c). Black rectangles indicate approximate positions of sequence variations of *p1* and *orf6* genes using type 2 strain FH as reference. Probable origins of the variation of these sequences (suffixes of RepMPs are identical to the variations) are indicated next to gray rectangles (also see panel B). Representative strains that harbor genes of the corresponding subtype are indicated on the left. Accession numbers of the subtype sequences are shown on the right. Type 2b is also referred to as type 2V in other reports [26,49,50]. Sequence information of the *orf6* gene of the type 2c2 strain P53 was kindly provided by Dr. Fei Zhao of the Chinese Center for Disease Control and Prevention. The *orf6* sequence of the type 2e strain Mp100 has not yet been reported [51]. (B) Distribution of RepMP regions in *M*. *pneumoniae* genome. Approximate positions of 8 RepMP4, 12 RepMP2/3, and 8 RepMP5 regions in *M*. *pneumoniae* genome are indicated by colored boxes. Two RepMP2/3 regions (RepMP2/3-k and -l) were newly identified during genome sequencing analysis of KCH-402 and KCH-405 strains (GenBank accession nos. AP017318 and AP017319) [25]. Suffixes of RepMP regions (-a to -k) are based on the nomenclature system proposed by Spuesens *et al*. previously [24,45].
(TIF)

S2 Fig Schematic illustration of the *p1* operon regions of FH and M241 strains. The light blue arrows indicate the *p1* and *orf6* genes. The *p1* gene of strain M241 had a truncation at the C-terminus due to DNA recombination between the repetitive sequence regions RepMP2/3-d and RepMP2/3_e (also see S1B Fig). Small orange arrows indicate approximate positions of PCR primer binding sites (ADH1, ADH2, ADH3, ADH4, and 23e-R1). The lower right panel is an electrophoresis pattern in 0.8% agarose gel of DNA fragments obtained as PCR products from FH and M241 strain genomes by using ADH3 and 23e-R1 primers. The regions corresponding to the PCR products are indicated by red arrows. A faint 1.9 kb band in FH strain suggests a presence of similar recombination event in growth population of FH strain.
(TIF)

S3 Fig The electrophoresis image of the PCR-RFLP analysis (Fig 2). (TIF)

S1 Table The values used to build the graphs of Fig 1. (PDF)

S2 Table Oligonucleotide Primers used for sequencing of *p1* operon region. (DOCX)

S3 Table The list of *M*. *pneumoniae* isolates collected and analyzed in this study. (PDF)

S4 Table The values used to build the graph of Fig 4. (PDF)
