# Supplementary figures and images for: Genetic characterization of Mycoplasma pneumoniae isolated in Osaka between 2011 and 2017: Decreased detection rate of macrolide-resistance and increase of p1 gene type 2 lineage strains
Source: PLoS One. 2019 Jan 25;14(1):e0209938. doi: 10.1371/journal.pone.0209938 (PMC6347185; doi:10.1371/journal.pone.0209938)

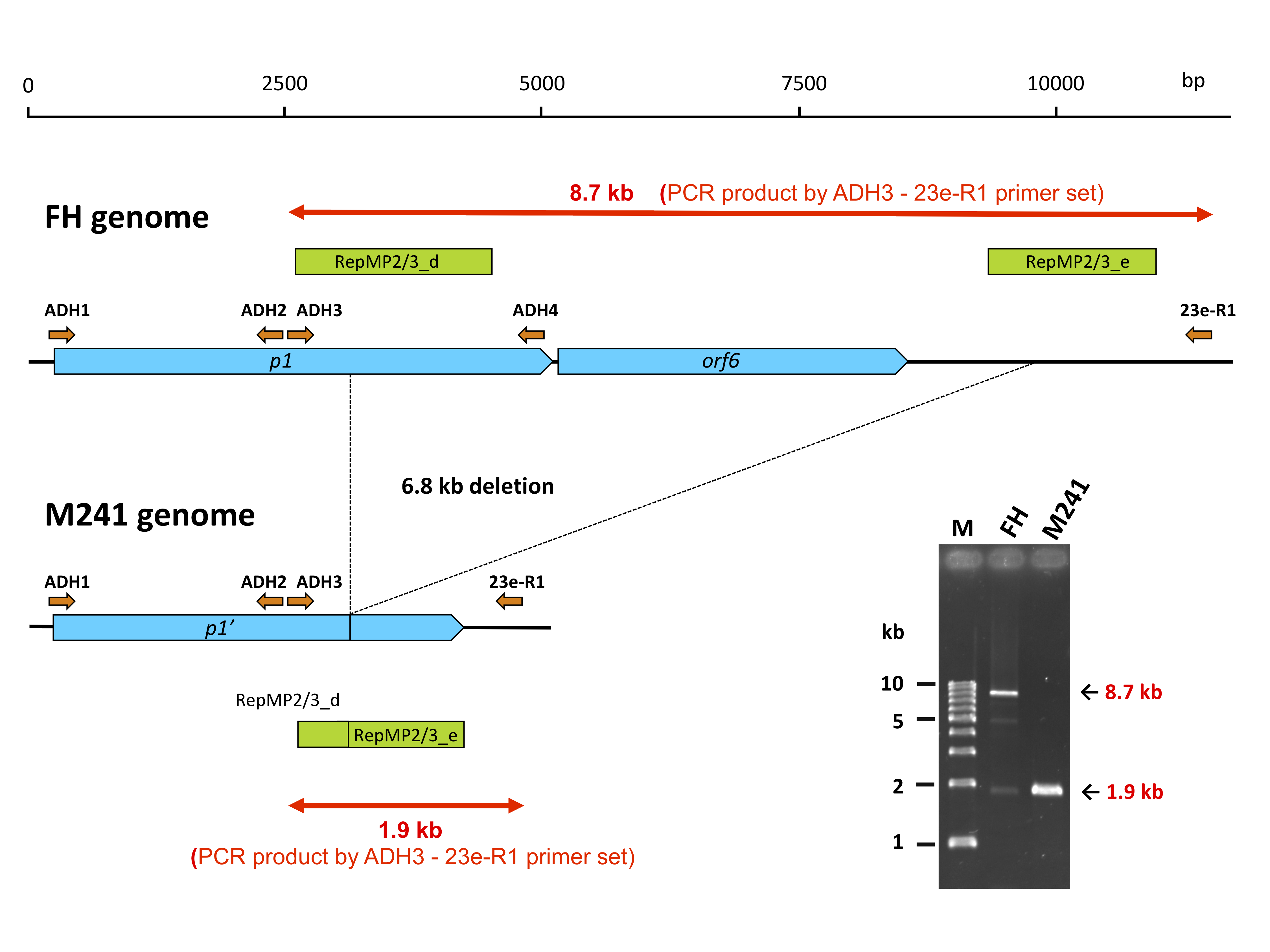

Supplement: S2 Fig — The light blue arrows indicate the p1 and orf6 genes. The p1 gene of strain M241 had a truncation at the C-terminus due to DNA recombination between the repetitive sequence regions RepMP2/3-d and RepMP2/3_e (also see S1B Fig). Small orange arrows indicate approximate positions of PCR primer binding sites (ADH1, ADH2, ADH3, ADH4, and 23e-R1). The lower right panel is an electrophoresis pattern in 0.8% agarose gel of DNA fragments obtained as PCR products from FH and M241 strain genomes by using ADH3 and 23e-R1 primers. The regions corresponding to the PCR products are indicated by red arrows. A faint 1.9 kb band in FH strain suggests a presence of similar recombination event in growth population of FH strain. (TIF) [file pone.0209938.s002.tif]

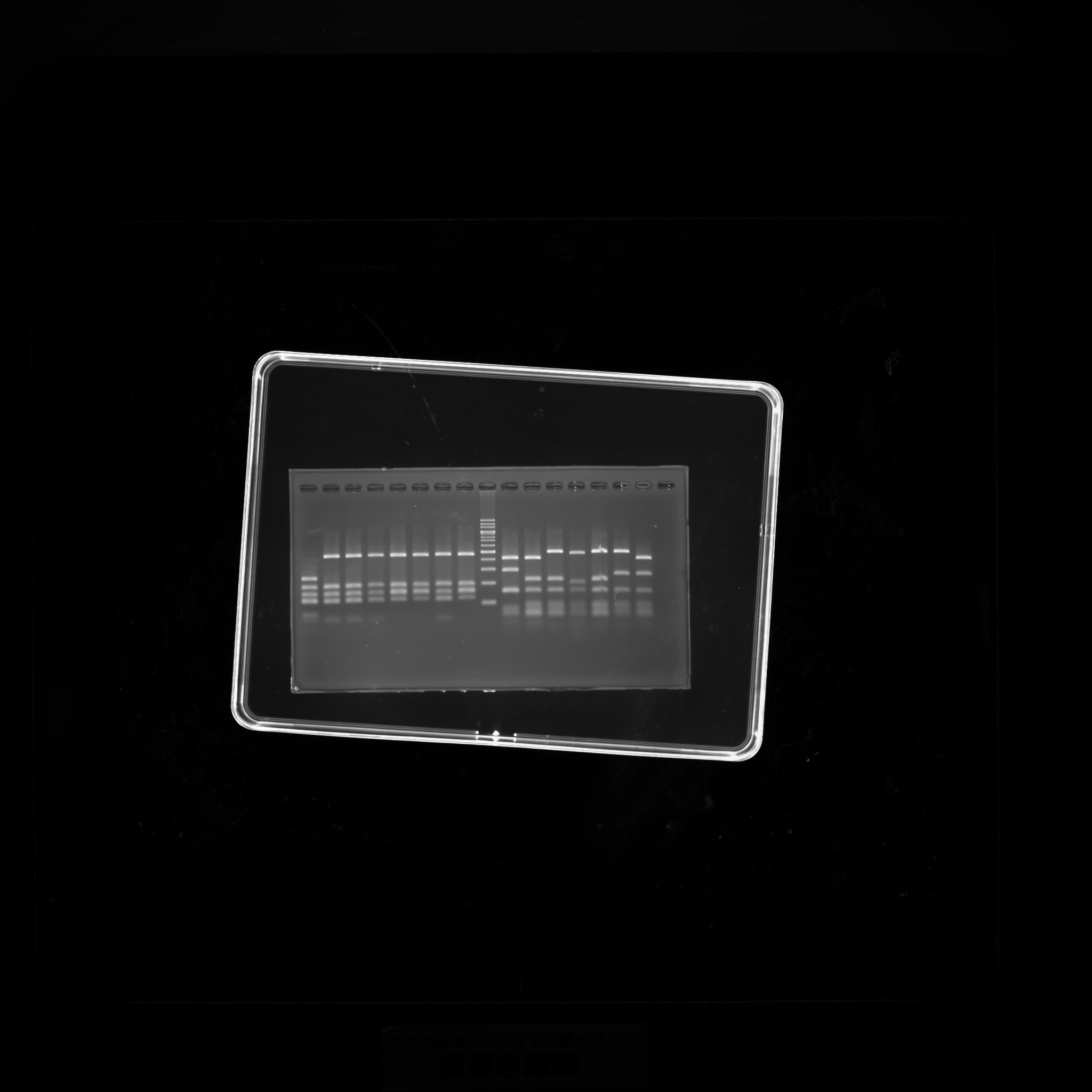

Supplement: S3 Fig — (TIF) [file pone.0209938.s003.tif]
