# Supplementary material for: Genetic characterization of Mycoplasma pneumoniae isolated in Osaka between 2011 and 2017: Decreased detection rate of macrolide-resistance and increase of p1 gene type 2 lineage strains
Source: PLoS One. 2019 Jan 25;14(1):e0209938. doi: 10.1371/journal.pone.0209938 (PMC6347185; doi:10.1371/journal.pone.0209938)
Supplement: S1 Table — (PDF) [file pone.0209938.s004.pdf]

S1 Table. The values used to build the graphs of Fig 1.

Year	Week	Patient number per sentinel		Number of sentinels	
		Nation avarage	Osaka	Nation	Osaka
1999	1				
1999	2				
1999	3				
1999	4				
1999	5				
1999	6				
1999	7				
1999	8				
1999	9				
1999	10				
1999	11				
1999	12				
1999	13				
1999	14	0.04	0.00	435	8
1999	15	0.04	0.00	436	7
1999	16	0.02	0.00	443	8
1999	17	0.04	0.00	446	8
1999	18	0.01	0.00	437	8
1999	19	0.09	0.00	435	7
1999	20	0.07	0.13	450	8
1999	21	0.08	0.00	453	8
1999	22	0.07	0.00	440	8
1999	23	0.05	0.00	451	8
1999	24	0.07	0.00	449	8
1999	25	0.05	0.13	458	8
1999	26	0.05	0.00	465	8
1999	27	0.05	0.00	454	8
1999	28	0.06	0.00	460	8
1999	29	0.04	0.00	456	8
1999	30	0.08	0.00	461	8
1999	31	0.07	0.00	463	8
1999	32	0.06	0.00	461	8
1999	33	0.06	0.00	465	8
1999	34	0.07	0.00	463	8
1999	35	0.08	0.00	460	8
1999	36	0.05	0.13	460	8
1999	37	0.06	0.00	471	8
1999	38	0.07	0.00	459	8
1999	39	0.06	0.00	472	7
1999	40	0.05	0.00	463	10
1999	41	0.08	0.00	460	8
1999	42	0.07	0.13	463	8
1999	43	0.07	0.00	465	8
1999	44	0.07	0.13	462	8
1999	45	0.11	0.13	463	8
1999	46	0.07	0.00	464	8
1999	47	0.11	0.00	460	8
1999	48	0.07	0.00	461	8
1999	49	0.11	0.00	457	8
1999	50	0.08	0.00	463	8
1999	51	0.08	0.14	460	7
1999	52	0.06	0.00	466	7
2000	1	0.07	0.00	457	8
2000	2	0.06	0.00	455	8
2000	3	0.06	0.00	452	8
2000	4	0.09	0.00	453	8
2000	5	0.06	0.00	458	8
2000	6	0.04	0.00	456	8
2000	7	0.07	0.00	458	8
2000	8	0.06	0.00	458	8
2000	9	0.07	0.00	457	8
2000	10	0.09	0.00	460	8
2000	11	0.05	0.00	457	8
2000	12	0.04	0.00	454	8
2000	13	0.05	0.13	456	8
2000	14	0.05	0.00	456	8
2000	15	0.05	0.00	462	8
2000	16	0.04	0.00	462	8
2000	17	0.06	0.00	463	8
2000	18	0.02	0.00	463	8
2000	19	0.04	0.00	462	8
2000	20	0.06	0.00	461	8
2000	21	0.07	0.00	463	8
2000	22	0.08	0.00	463	8
2000	23	0.07	0.00	464	8
2000	24	0.11	0.00	465	8
2000	25	0.06	0.00	464	8
2000	26	0.07	0.13	459	8
2000	27	0.09	0.00	464	8
2000	28	0.08	0.00	462	8
2000	29	0.05	0.00	463	8
2000	30	0.08	0.00	465	8
2000	31	0.10	0.00	461	8
2000	32	0.08	0.00	460	8
2000	33	0.08	0.00	458	8
2000	34	0.09	0.25	461	8
2000	35	0.07	0.00	460	8
2000	36	0.08	0.25	460	8
2000	37	0.06	0.00	461	8
2000	38	0.08	0.13	461	8
2000	39	0.10	0.00	462	8
2000	40	0.12	0.25	459	8
2000	41	0.11	0.00	454	8
2000	42	0.13	0.00	461	8
2000	43	0.19	0.00	461	8
2000	44	0.10	0.00	461	8
2000	45	0.14	0.00	461	8
2000	46	0.16	0.25	461	8
2000	47	0.16	0.00	462	8
2000	48	0.21	0.13	461	8
2000	49	0.16	0.25	461	8
2000	50	0.16	0.38	462	8
2000	51	0.18	0.00	461	8
2000	52	0.13	0.00	454	8
2001	1	0.07	0.00	466	9
2001	2	0.13	0.00	466	9
2001	3	0.14	0.00	466	9
2001	4	0.12	0.00	467	9
2001	5	0.16	0.00	467	9
2001	6	0.12	0.11	467	9
2001	7	0.09	0.00	467	9
2001	8	0.13	0.00	465	9
2001	9	0.11	0.00	466	9
2001	10	0.12	0.00	466	9
2001	11	0.10	0.11	466	9
2001	12	0.08	0.11	467	9
2001	13	0.12	0.00	466	9
2001	14	0.11	0.15	470	13
2001	15	0.14	0.77	471	13
2001	16	0.12	0.23	471	13
2001	17	0.09	0.15	470	13
2001	18	0.08	0.29	473	14
2001	19	0.14	0.14	473	14
2001	20	0.15	0.36	473	14
2001	21	0.12	0.14	472	14
2001	22	0.13	0.57	473	14
2001	23	0.13	0.07	473	14
2001	24	0.13	0.00	473	14
2001	25	0.14	0.21	473	14
2001	26	0.12	0.00	473	14
2001	27	0.16	0.43	473	14
2001	28	0.17	0.21	473	14
2001	29	0.16	0.21	473	14
2001	30	0.13	0.50	472	14
2001	31	0.20	0.64	473	14
2001	32	0.18	0.57	472	14
2001	33	0.16	0.57	472	14
2001	34	0.18	0.29	472	14
2001	35	0.18	0.36	472	14
2001	36	0.24	0.79	472	14
2001	37	0.18	0.29	474	14
2001	38	0.17	0.36	472	14

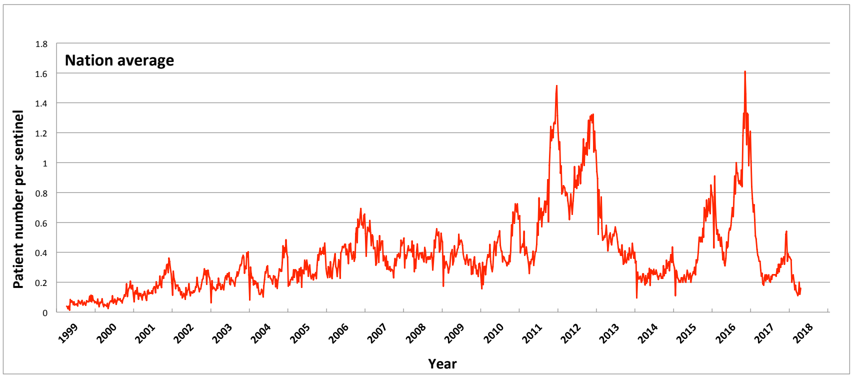

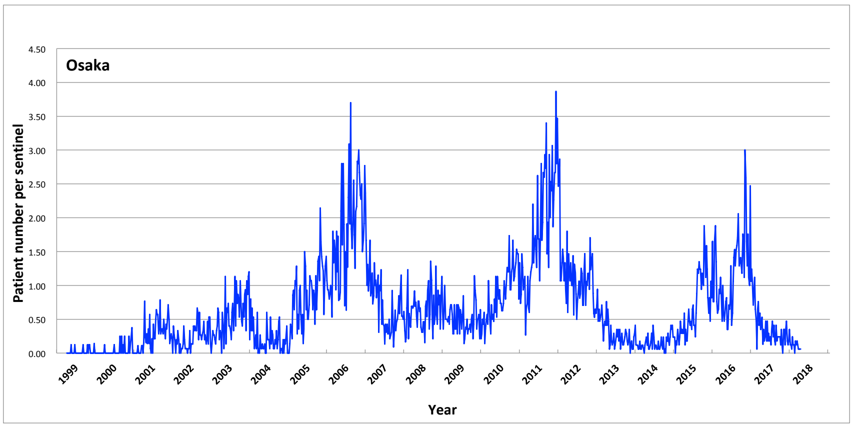

2001	39	0.24	0.36	471	14
2001	40	0.24	0.29	472	14
2001	41	0.23	0.50	470	14
2001	42	0.28	0.36	471	14
2001	43	0.25	0.21	469	14
2001	44	0.26	0.43	471	14
2001	45	0.32	0.36	472	14
2001	46	0.32	0.50	472	14
2001	47	0.26	0.71	472	14
2001	48	0.36	0.50	472	14
2001	49	0.34	0.14	472	14
2001	50	0.30	0.21	472	14
2001	51	0.25	0.29	473	14
2001	52	0.19	0.21	473	14
2002	1	0.11	0.00	473	15
2002	2	0.27	0.40	474	15
2002	3	0.23	0.27	474	15
2002	4	0.20	0.20	474	15
2002	5	0.16	0.00	472	15
2002	6	0.18	0.20	472	15
2002	7	0.18	0.13	474	15
2002	8	0.18	0.33	474	15
2002	9	0.14	0.20	473	15
2002	10	0.16	0.00	474	15
2002	11	0.15	0.13	474	15
2002	12	0.12	0.00	474	15
2002	13	0.14	0.07	473	15
2002	14	0.09	0.07	474	15
2002	15	0.10	0.13	474	15
2002	16	0.13	0.40	474	15
2002	17	0.12	0.20	474	15
2002	18	0.09	0.07	474	15
2002	19	0.16	0.07	475	15
2002	20	0.12	0.07	475	15
2002	21	0.14	0.00	475	15
2002	22	0.15	0.07	475	15
2002	23	0.19	0.07	475	15
2002	24	0.13	0.33	475	15
2002	25	0.11	0.00	475	15
2002	26	0.13	0.13	475	15
2002	27	0.13	0.13	475	15
2002	28	0.13	0.13	475	15
2002	29	0.14	0.40	474	15
2002	30	0.13	0.33	474	15
2002	31	0.15	0.40	475	15
2002	32	0.17	0.33	474	15
2002	33	0.17	0.33	474	15
2002	34	0.23	0.67	473	15
2002	35	0.19	0.60	474	15
2002	36	0.16	0.20	474	15
2002	37	0.14	0.60	474	15
2002	38	0.16	0.27	474	15
2002	39	0.16	0.27	474	15
2002	40	0.18	0.20	474	15
2002	41	0.22	0.27	474	15
2002	42	0.22	0.33	474	15
2002	43	0.25	0.47	473	15
2002	44	0.29	0.20	474	15
2002	45	0.25	0.27	473	15
2002	46	0.28	0.13	473	15
2002	47	0.19	0.20	473	15
2002	48	0.28	0.47	474	15
2002	49	0.28	0.13	474	15
2002	50	0.24	0.53	473	15
2002	51	0.23	0.53	473	15
2002	52	0.19	0.13	474	15
2003	1	0.06	0.00	465	15
2003	2	0.21	0.13	468	15
2003	3	0.15	0.33	471	15
2003	4	0.17	0.27	472	15
2003	5	0.16	0.27	472	15
2003	6	0.15	0.20	471	15
2003	7	0.14	0.13	472	15
2003	8	0.15	0.33	472	15
2003	9	0.18	0.40	471	15
2003	10	0.16	0.67	472	15
2003	11	0.23	0.60	471	15
2003	12	0.18	0.20	471	15
2003	13	0.19	0.33	471	15
2003	14	0.15	0.27	472	15
2003	15	0.15	0.00	471	15
2003	16	0.18	0.60	471	15
2003	17	0.17	0.40	471	15
2003	18	0.15	0.07	470	15
2003	19	0.20	0.40	471	15
2003	20	0.23	1.13	471	15
2003	21	0.25	0.13	473	15
2003	22	0.22	0.67	471	15
2003	23	0.26	0.60	471	15
2003	24	0.24	0.73	471	15
2003	25	0.27	0.40	471	15
2003	26	0.19	0.40	471	15
2003	27	0.18	0.33	471	15
2003	28	0.23	0.20	471	15
2003	29	0.21	0.60	471	15
2003	30	0.21	0.73	471	15
2003	31	0.20	0.33	471	15
2003	32	0.19	0.67	471	15
2003	33	0.17	1.13	471	15
2003	34	0.23	0.40	471	15
2003	35	0.24	1.07	471	15
2003	36	0.27	0.73	472	15
2003	37	0.24	0.87	472	15
2003	38	0.26	0.80	472	15
2003	39	0.29	0.53	472	15
2003	40	0.32	1.07	473	15
2003	41	0.31	0.73	473	15
2003	42	0.33	0.47	473	15
2003	43	0.36	0.40	473	15
2003	44	0.38	0.73	473	15
2003	45	0.37	0.53	473	15
2003	46	0.26	0.87	473	15
2003	47	0.31	0.93	473	15
2003	48	0.29	0.33	473	15
2003	49	0.40	1.07	473	15
2003	50	0.37	0.87	473	15
2003	51	0.40	1.13	473	15
2003	52	0.25	1.20	473	15
2004	1	0.08	0.20	501	15
2004	2	0.30	0.80	481	15
2004	3	0.26	0.33	478	15
2004	4	0.23	0.67	478	15
2004	5	0.23	0.33	479	15
2004	6	0.18	0.53	479	15
2004	7	0.21	0.07	479	15
2004	8	0.19	0.13	479	15
2004	9	0.21	0.27	479	15
2004	10	0.19	0.07	479	15
2004	11	0.16	0.60	479	15
2004	12	0.19	0.00	479	15
2004	13	0.15	0.07	475	15
2004	14	0.12	0.00	478	15
2004	15	0.12	0.27	479	15
2004	16	0.13	0.07	479	15
2004	17	0.15	0.07	475	15
2004	18	0.14	0.13	474	15
2004	19	0.10	0.07	474	15
2004	20	0.18	0.07	474	15
2004	21	0.22	0.13	476	15
2004	22	0.27	0.00	475	15
2004	23	0.26	0.00	474	15
2004	24	0.30	0.60	474	15
2004	25	0.31	0.20	474	15
2004	26	0.23	0.20	474	15
2004	27	0.24	0.20	474	15
2004	28	0.24	0.07	474	15

2004	29	0.22	0.53	473	15
2004	30	0.22	0.13	474	15
2004	31	0.21	0.33	473	15
2004	32	0.19	0.27	474	15
2004	33	0.24	0.07	464	15
2004	34	0.23	0.07	473	15
2004	35	0.24	0.20	473	15
2004	36	0.17	0.13	473	15
2004	37	0.22	0.33	473	15
2004	38	0.16	0.07	473	15
2004	39	0.21	0.20	473	15
2004	40	0.24	0.47	473	15
2004	41	0.29	0.53	472	15
2004	42	0.29	0.00	473	15
2004	43	0.27	0.07	473	15
2004	44	0.27	0.07	473	15
2004	45	0.33	0.00	473	15
2004	46	0.44	0.20	473	15
2004	47	0.36	0.00	472	15
2004	48	0.43	0.20	473	15
2004	49	0.40	0.13	473	15
2004	50	0.48	0.27	473	15
2004	51	0.39	0.40	473	15
2004	52	0.37	0.00	473	15
2004	53	0.17	0.00	471	15
2005	1	0.20	0.00	470	14
2005	2	0.18	0.14	470	14
2005	3	0.22	0.43	470	14
2005	4	0.22	0.36	470	14
2005	5	0.23	0.21	469	14
2005	6	0.22	0.86	469	14
2005	7	0.28	0.93	469	14
2005	8	0.23	0.64	469	14
2005	9	0.28	1.07	469	14
2005	10	0.28	1.00	469	14
2005	11	0.28	1.29	469	14
2005	12	0.20	0.29	469	14
2005	13	0.27	0.57	470	14
2005	14	0.20	0.36	472	14
2005	15	0.29	0.93	471	14
2005	16	0.24	1.00	472	14
2005	17	0.30	0.57	472	14
2005	18	0.17	0.29	471	14
2005	19	0.28	0.57	471	14
2005	20	0.28	0.14	471	14
2005	21	0.26	0.36	472	14
2005	22	0.35	1.50	472	14
2005	23	0.33	1.29	472	14
2005	24	0.28	0.64	472	14
2005	25	0.35	0.93	472	14
2005	26	0.31	0.50	472	14
2005	27	0.24	0.50	474	14
2005	28	0.29	0.71	472	14
2005	29	0.32	1.14	472	14
2005	30	0.24	1.07	472	14
2005	31	0.22	1.07	472	14
2005	32	0.24	0.93	472	14
2005	33	0.25	0.64	472	14
2005	34	0.22	0.93	472	14
2005	35	0.25	0.86	472	14
2005	36	0.24	0.43	472	14
2005	37	0.26	1.14	472	14
2005	38	0.22	0.64	472	14
2005	39	0.31	0.93	472	14
2005	40	0.39	1.36	472	14
2005	41	0.35	1.43	472	14
2005	42	0.39	1.07	472	14
2005	43	0.43	2.14	472	14
2005	44	0.39	1.64	472	14
2005	45	0.40	1.43	472	14
2005	46	0.39	1.29	472	14
2005	47	0.43	1.21	472	14
2005	48	0.39	0.57	472	14
2005	49	0.46	1.00	472	14
2005	50	0.39	1.29	472	14
2005	51	0.29	1.43	473	14
2005	52	0.26	1.07	470	14
2006	1	0.23	0.93	378	15
2006	2	0.30	0.93	384	15
2006	3	0.29	0.80	385	15
2006	4	0.23	0.87	383	15
2006	5	0.34	1.00	386	15
2006	6	0.24	0.93	384	15
2006	7	0.22	0.53	378	15
2006	8	0.29	1.80	386	15
2006	9	0.32	1.33	384	15
2006	10	0.35	1.67	382	15
2006	11	0.39	1.53	383	15
2006	12	0.30	0.93	383	15
2006	13	0.29	1.80	414	10
2006	14	0.24	1.20	436	10
2006	15	0.30	1.73	433	11
2006	16	0.35	0.78	441	9
2006	17	0.34	0.80	441	10
2006	18	0.23	0.90	442	10
2006	19	0.40	2.10	445	10
2006	20	0.41	2.80	447	10
2006	21	0.44	1.60	449	10
2006	22	0.44	2.80	451	10
2006	23	0.43	1.90	452	10
2006	24	0.45	0.70	453	10
2006	25	0.45	1.50	449	10
2006	26	0.38	0.64	453	11
2006	27	0.42	1.91	450	11
2006	28	0.40	1.27	452	11
2006	29	0.33	2.30	452	10
2006	30	0.45	3.09	451	11
2006	31	0.40	1.91	452	11
2006	32	0.46	3.70	451	10
2006	33	0.40	1.83	453	12
2006	34	0.35	1.55	452	11
2006	35	0.37	2.00	452	11
2006	36	0.41	2.56	451	9
2006	37	0.34	1.75	452	12
2006	38	0.37	1.25	453	12
2006	39	0.52	2.09	452	11
2006	40	0.56	2.17	450	12
2006	41	0.46	2.83	454	12
2006	42	0.62	2.80	451	10
2006	43	0.57	3.00	453	12
2006	44	0.59	2.67	454	12
2006	45	0.65	2.33	454	12
2006	46	0.69	2.27	456	11
2006	47	0.58	2.50	456	12
2006	48	0.60	1.50	455	12
2006	49	0.56	1.67	453	12
2006	50	0.65	1.92	454	12
2006	51	0.66	2.77	456	13
2006	52	0.53	2.36	451	11
2007	1	0.37	1.67	455	12
2007	2	0.48	1.00	454	13
2007	3	0.57	1.31	457	13
2007	4	0.55	0.92	455	12
2007	5	0.54	1.08	454	12
2007	6	0.61	1.67	455	12
2007	7	0.53	0.85	457	13
2007	8	0.45	0.92	456	12
2007	9	0.52	1.18	454	11
2007	10	0.51	0.73	454	11
2007	11	0.42	0.85	457	13
2007	12	0.43	1.33	453	12
2007	13	0.43	1.00	455	13
2007	14	0.31	1.08	455	13
2007	15	0.33	0.82	453	11
2007	16	0.48	1.00	457	13
2007	17	0.41	1.15	457	13

2007	18	0.33	0.31	458	13
2007	19	0.51	1.00	457	13
2007	20	0.41	0.92	459	13
2007	21	0.44	1.23	459	13
2007	22	0.48	0.43	462	14
2007	23	0.48	0.79	464	14
2007	24	0.34	0.43	466	14
2007	25	0.38	0.40	466	15
2007	26	0.36	0.13	468	15
2007	27	0.31	0.43	465	14
2007	28	0.38	0.31	465	13
2007	29	0.29	0.43	467	14
2007	30	0.34	0.29	467	14
2007	31	0.29	0.29	465	14
2007	32	0.28	0.50	463	14
2007	33	0.31	0.21	465	14
2007	34	0.27	0.27	467	15
2007	35	0.29	0.31	464	13
2007	36	0.28	0.92	464	13
2007	37	0.28	0.54	464	13
2007	38	0.23	0.09	463	11
2007	39	0.31	0.36	462	11
2007	40	0.36	0.64	462	11
2007	41	0.31	0.33	463	12
2007	42	0.35	0.46	465	13
2007	43	0.38	0.50	462	12
2007	44	0.37	0.62	465	13
2007	45	0.39	0.85	464	13
2007	46	0.36	0.58	462	12
2007	47	0.38	0.85	463	13
2007	48	0.48	1.15	464	13
2007	49	0.44	0.55	462	11
2007	50	0.46	0.55	461	11
2007	51	0.48	0.50	463	12
2007	52	0.50	0.62	460	13
2008	1	0.30	0.33	464	13
2008	2	0.38	0.17	462	12
2008	3	0.36	0.23	466	13
2008	4	0.38	0.67	460	12
2008	5	0.42	1.23	463	13
2008	6	0.44	0.21	465	14
2008	7	0.41	0.58	462	12
2008	8	0.41	0.46	464	13
2008	9	0.42	0.38	464	13
2008	10	0.40	0.69	465	13
2008	11	0.48	0.69	466	13
2008	12	0.37	0.67	464	12
2008	13	0.45	0.69	462	13
2008	14	0.27	0.92	460	12
2008	15	0.35	0.69	461	13
2008	16	0.29	0.75	461	12
2008	17	0.41	0.54	465	13
2008	18	0.46	0.38	461	13
2008	19	0.38	0.77	462	13
2008	20	0.35	0.77	462	13
2008	21	0.39	0.62	462	13
2008	22	0.39	0.67	464	12
2008	23	0.40	0.38	465	13
2008	24	0.36	0.31	463	13
2008	25	0.40	0.31	464	13
2008	26	0.35	0.46	464	13
2008	27	0.34	0.46	464	13
2008	28	0.43	0.15	464	13
2008	29	0.41	0.69	463	13
2008	30	0.33	0.77	465	13
2008	31	0.36	0.54	465	13
2008	32	0.37	0.93	465	14
2008	33	0.35	1.07	465	14
2008	34	0.37	0.36	463	14
2008	35	0.35	0.57	463	14
2008	36	0.37	1.36	463	14
2008	37	0.40	1.08	463	13
2008	38	0.36	0.50	466	14
2008	39	0.28	0.21	465	14
2008	40	0.37	0.86	465	14
2008	41	0.48	0.43	465	14
2008	42	0.43	0.57	466	14
2008	43	0.51	1.29	465	14
2008	44	0.56	0.57	464	14
2008	45	0.51	0.43	465	14
2008	46	0.51	0.93	466	14
2008	47	0.54	0.71	464	14
2008	48	0.40	0.86	465	14
2008	49	0.42	0.36	466	14
2008	50	0.53	0.80	464	15
2008	51	0.48	1.13	466	15
2008	52	0.47	0.62	462	13
2009	1	0.17	0.36	465	14
2009	2	0.38	0.64	465	14
2009	3	0.30	1.00	467	14
2009	4	0.33	0.50	466	14
2009	5	0.34	0.50	467	14
2009	6	0.32	0.50	467	14
2009	7	0.30	0.46	464	13
2009	8	0.26	0.54	466	13
2009	9	0.29	0.43	466	14
2009	10	0.31	0.64	466	14
2009	11	0.39	0.71	466	14
2009	12	0.40	0.71	467	14
2009	13	0.42	0.57	465	14
2009	14	0.32	0.29	466	14
2009	15	0.34	0.29	464	14
2009	16	0.44	0.71	465	14
2009	17	0.39	0.36	465	14
2009	18	0.37	0.36	465	14
2009	19	0.39	0.71	463	14
2009	20	0.42	0.29	463	14
2009	21	0.43	0.71	463	14
2009	22	0.52	0.67	461	12
2009	23	0.45	0.29	465	14
2009	24	0.48	0.64	466	14
2009	25	0.48	0.57	464	14
2009	26	0.47	0.36	465	14
2009	27	0.45	0.57	463	14
2009	28	0.39	0.85	467	13
2009	29	0.36	0.14	464	14
2009	30	0.35	0.21	465	14
2009	31	0.32	0.38	463	13
2009	32	0.36	0.36	464	14
2009	33	0.38	0.50	467	14
2009	34	0.33	0.29	466	14
2009	35	0.31	0.36	465	14
2009	36	0.35	0.36	466	14
2009	37	0.35	0.43	466	14
2009	38	0.35	0.31	462	13
2009	39	0.25	0.50	464	14
2009	40	0.30	0.57	465	14
2009	41	0.26	0.21	464	14
2009	42	0.34	0.43	462	14
2009	43	0.37	1.14	462	14
2009	44	0.30	0.93	461	14
2009	45	0.31	0.79	464	14
2009	46	0.25	0.36	463	14
2009	47	0.28	0.50	464	14
2009	48	0.24	0.50	462	14
2009	49	0.32	0.57	465	14
2009	50	0.33	0.21	463	14
2009	51	0.30	0.64	464	14
2009	52	0.21	0.21	464	14
2009	53	0.16	0.29	465	14
2010	1	0.33	0.50	464	14
2010	2	0.18	0.43	466	14
2010	3	0.30	0.60	465	15
2010	4	0.31	0.14	466	14
2010	5	0.25	0.36	466	14
2010	6	0.31	0.50	462	14

2010	7	0.35	0.57	463	14
2010	8	0.39	0.43	464	14
2010	9	0.37	1.07	463	14
2010	10	0.38	0.71	464	14
2010	11	0.45	0.43	466	14
2010	12	0.34	0.29	464	14
2010	13	0.29	0.71	465	14
2010	14	0.27	0.50	464	14
2010	15	0.30	0.57	462	14
2010	16	0.40	0.47	462	15
2010	17	0.47	0.80	463	15
2010	18	0.42	0.60	465	15
2010	19	0.38	1.13	464	15
2010	20	0.47	0.53	465	15
2010	21	0.46	1.13	463	15
2010	22	0.50	0.93	464	15
2010	23	0.51	0.80	463	15
2010	24	0.54	0.93	463	15
2010	25	0.45	0.73	465	15
2010	26	0.41	0.73	463	15
2010	27	0.41	0.63	463	16
2010	28	0.34	0.63	464	16
2010	29	0.37	0.73	461	15
2010	30	0.37	1.07	462	15
2010	31	0.35	0.93	462	15
2010	32	0.34	0.80	461	15
2010	33	0.33	1.00	460	15
2010	34	0.31	1.27	461	15
2010	35	0.33	1.20	460	15
2010	36	0.32	1.20	462	15
2010	37	0.38	1.73	461	15
2010	38	0.34	0.93	463	15
2010	39	0.41	0.93	465	15
2010	40	0.58	1.20	462	15
2010	41	0.51	1.27	464	15
2010	42	0.67	1.67	463	15
2010	43	0.61	1.07	466	15
2010	44	0.59	1.13	464	15
2010	45	0.72	1.27	465	15
2010	46	0.71	1.33	465	15
2010	47	0.67	1.53	464	15
2010	48	0.72	1.47	465	15
2010	49	0.62	1.47	463	15
2010	50	0.63	0.80	464	15
2010	51	0.65	1.33	463	15
2010	52	0.40	0.73	463	15
2011	1	0.43	0.87	467	15
2011	2	0.44	1.47	467	15
2011	3	0.46	1.27	468	15
2011	4	0.44	0.93	468	15
2011	5	0.54	1.07	467	15
2011	6	0.38	1.13	465	15
2011	7	0.39	0.27	465	15
2011	8	0.44	0.67	466	15
2011	9	0.39	0.73	457	15
2011	10	0.36	1.13	455	15
2011	11	0.32	0.67	457	15
2011	12	0.26	0.60	459	15
2011	13	0.34	1.07	454	15
2011	14	0.32	1.20	460	15
2011	15	0.35	1.27	460	15
2011	16	0.35	1.33	461	15
2011	17	0.41	2.20	463	15
2011	18	0.31	1.33	463	15
2011	19	0.35	1.67	464	15
2011	20	0.40	1.73	465	15
2011	21	0.41	1.53	465	15
2011	22	0.45	1.27	465	16
2011	23	0.60	2.63	466	16
2011	24	0.52	1.69	467	16
2011	25	0.76	1.69	466	15
2011	26	0.62	1.07	465	15
2011	27	0.65	1.67	466	15
2011	28	0.70	2.80	466	15
2011	29	0.57	1.67	466	15
2011	30	0.67	1.93	466	15
2011	31	0.65	2.67	464	15
2011	32	0.69	2.60	467	15
2011	33	0.74	2.93	465	15
2011	34	0.67	2.73	465	15
2011	35	0.74	3.40	465	15
2011	36	0.72	1.47	466	15
2011	37	0.89	1.93	465	15
2011	38	0.60	1.27	466	15
2011	39	0.98	2.93	466	15
2011	40	1.10	2.00	464	15
2011	41	1.24	2.53	467	15
2011	42	1.15	2.40	466	15
2011	43	1.22	3.07	467	15
2011	44	1.17	1.87	467	15
2011	45	1.26	2.27	468	15
2011	46	1.27	2.67	468	15
2011	47	1.26	2.67	468	15
2011	48	1.46	3.87	468	15
2011	49	1.51	2.80	468	15
2011	50	1.31	3.47	468	15
2011	51	1.20	2.47	467	15
2011	52	1.09	2.67	464	15
2012	1	1.14	2.87	467	15
2012	2	0.93	1.07	467	15
2012	3	0.98	1.07	467	15
2012	4	0.79	1.33	467	15
2012	5	0.81	1.53	467	15
2012	6	0.85	1.40	467	15
2012	7	0.84	1.07	468	15
2012	8	0.83	1.33	466	15
2012	9	0.78	0.93	468	15
2012	10	0.80	0.73	466	15
2012	11	0.79	1.80	466	15
2012	12	0.75	0.60	467	15
2012	13	0.71	1.47	467	15
2012	14	0.62	1.20	468	15
2012	15	0.72	1.07	467	15
2012	16	0.79	1.33	467	15
2012	17	0.72	1.00	466	15
2012	18	0.65	1.00	466	15
2012	19	0.74	1.44	468	16
2012	20	0.81	0.56	469	16
2012	21	0.97	1.31	468	16
2012	22	0.84	1.19	469	16
2012	23	0.88	0.88	469	16
2012	24	0.83	0.50	469	16
2012	25	0.91	0.75	470	16
2012	26	0.85	0.88	468	16
2012	27	0.98	1.06	468	16
2012	28	0.95	0.81	467	16
2012	29	0.86	0.88	468	16
2012	30	0.99	1.25	469	16
2012	31	0.95	1.18	469	17
2012	32	1.00	0.71	469	17
2012	33	1.16	0.71	468	17
2012	34	0.98	1.24	469	17
2012	35	1.10	1.47	470	17
2012	36	1.06	0.71	468	17
2012	37	1.13	1.06	468	17
2012	38	1.05	1.00	468	17
2012	39	1.20	1.18	466	17
2012	40	1.28	1.18	469	17
2012	41	1.09	0.94	470	17
2012	42	1.31	1.71	469	17
2012	43	1.28	1.24	470	17
2012	44	1.32	1.35	468	17
2012	45	1.27	1.47	468	17
2012	46	1.32	0.88	469	17
2012	47	1.07	0.59	469	17
2012	48	1.21	0.53	468	17

2012	49	1.08	0.65	469	17
2012	50	1.08	0.59	469	17
2012	51	0.94	0.71	468	17
2012	52	0.89	0.94	468	17
2013	1	0.52	0.47	468	17
2013	2	0.82	0.65	467	17
2013	3	0.65	0.47	471	17
2013	4	0.63	0.53	471	17
2013	5	0.77	0.59	470	17
2013	6	0.56	0.71	470	17
2013	7	0.48	0.41	470	17
2013	8	0.49	0.18	469	17
2013	9	0.51	0.41	471	17
2013	10	0.50	0.47	470	17
2013	11	0.53	0.41	470	17
2013	12	0.58	0.76	470	17
2013	13	0.48	0.41	467	17
2013	14	0.41	0.65	472	17
2013	15	0.46	0.47	472	17
2013	16	0.51	0.06	471	17
2013	17	0.53	0.12	474	17
2013	18	0.47	0.41	472	17
2013	19	0.45	0.18	471	17
2013	20	0.49	0.12	472	17
2013	21	0.52	0.18	473	17
2013	22	0.51	0.29	474	17
2013	23	0.54	0.06	474	17
2013	24	0.57	0.12	473	17
2013	25	0.54	0.24	473	17
2013	26	0.52	0.35	471	17
2013	27	0.47	0.24	473	17
2013	28	0.43	0.35	473	17
2013	29	0.40	0.18	473	17
2013	30	0.42	0.35	473	17
2013	31	0.38	0.06	473	17
2013	32	0.38	0.18	472	17
2013	33	0.45	0.29	473	17
2013	34	0.38	0.12	472	17
2013	35	0.32	0.12	473	17
2013	36	0.33	0.18	473	17
2013	37	0.41	0.24	473	17
2013	38	0.34	0.35	470	17
2013	39	0.33	0.29	472	17
2013	40	0.36	0.18	472	17
2013	41	0.39	0.12	471	17
2013	42	0.35	0.18	471	17
2013	43	0.43	0.35	472	17
2013	44	0.39	0.00	472	17
2013	45	0.36	0.12	472	17
2013	46	0.39	0.18	471	17
2013	47	0.46	0.06	472	17
2013	48	0.41	0.06	472	17
2013	49	0.36	0.24	471	17
2013	50	0.40	0.29	473	17
2013	51	0.33	0.41	472	17
2013	52	0.34	0.12	473	17
2014	1	0.10	0.12	473	17
2014	2	0.27	0.12	474	17
2014	3	0.22	0.06	474	17
2014	4	0.22	0.06	475	17
2014	5	0.24	0.18	475	17
2014	6	0.24	0.06	475	17
2014	7	0.20	0.06	474	17
2014	8	0.21	0.12	473	17
2014	9	0.26	0.12	474	17
2014	10	0.26	0.06	476	17
2014	11	0.22	0.00	475	17
2014	12	0.18	0.12	475	17
2014	13	0.24	0.18	475	17
2014	14	0.19	0.24	475	17
2014	15	0.20	0.12	474	17
2014	16	0.26	0.29	476	17
2014	17	0.23	0.06	475	17
2014	18	0.29	0.06	474	17
2014	19	0.18	0.12	475	17
2014	20	0.28	0.18	476	17
2014	21	0.26	0.29	476	17
2014	22	0.28	0.06	476	17
2014	23	0.25	0.41	475	17
2014	24	0.29	0.24	475	17
2014	25	0.25	0.06	476	17
2014	26	0.25	0.12	476	17
2014	27	0.28	0.12	475	17
2014	28	0.26	0.06	476	17
2014	29	0.24	0.06	476	17
2014	30	0.22	0.18	476	17
2014	31	0.24	0.06	475	17
2014	32	0.24	0.06	474	17
2014	33	0.26	0.12	475	17
2014	34	0.23	0.24	476	17
2014	35	0.24	0.12	476	17
2014	36	0.23	0.06	476	17
2014	37	0.31	0.24	476	17
2014	38	0.24	0.00	474	17
2014	39	0.29	0.06	476	17
2014	40	0.26	0.00	474	17
2014	41	0.35	0.24	476	17
2014	42	0.29	0.18	475	17
2014	43	0.32	0.29	476	17
2014	44	0.34	0.18	475	17
2014	45	0.28	0.12	476	17
2014	46	0.34	0.24	476	17
2014	47	0.39	0.41	475	17
2014	48	0.33	0.35	476	17
2014	49	0.44	0.41	475	17
2014	50	0.34	0.24	475	17
2014	51	0.31	0.24	474	17
2014	52	0.29	0.24	473	17
2015	1	0.11	0	476	17
2015	2	0.28	0.18	477	17
2015	3	0.27	0.12	477	17
2015	4	0.23	0.35	477	17
2015	5	0.22	0.24	478	17
2015	6	0.2	0.12	478	17
2015	7	0.21	0.24	478	17
2015	8	0.2	0.47	478	17
2015	9	0.23	0.29	478	17
2015	10	0.23	0.35	478	17
2015	11	0.2	0.29	478	17
2015	12	0.23	0.29	478	17
2015	13	0.25	0.59	478	17
2015	14	0.21	0.12	478	17
2015	15	0.2	0.35	478	17
2015	16	0.24	0.18	478	17
2015	17	0.28	0.18	478	17
2015	18	0.26	0.47	477	17
2015	19	0.22	0.65	478	17
2015	20	0.25	0.47	478	17
2015	21	0.26	0.41	476	17
2015	22	0.2	0.47	478	17
2015	23	0.24	0.35	478	17
2015	24	0.25	0.65	478	17
2015	25	0.27	0.71	478	17
2015	26	0.26	0.41	477	17
2015	27	0.3	0.41	478	17
2015	28	0.25	0.24	478	17
2015	29	0.35	0.59	478	17
2015	30	0.33	0.88	478	17
2015	31	0.35	1.24	478	17
2015	32	0.4	1.18	476	17
2015	33	0.5	1.35	478	17
2015	34	0.5	1.24	478	17
2015	35	0.47	1.24	478	17
2015	36	0.5	0.94	478	17
2015	37	0.47	1.18	477	17
2015	38	0.56	1.06	477	17

2015	39	0.5	1	478	17
2015	40	0.7	1.88	477	17
2015	41	0.68	1.47	477	17
2015	42	0.61	1.12	478	17
2015	43	0.75	1.59	477	17
2015	44	0.64	1.24	478	17
2015	45	0.68	0.82	478	17
2015	46	0.76	0.59	477	17
2015	47	0.68	0.82	477	17
2015	48	0.66	0.47	477	17
2015	49	0.85	1.06	478	17
2015	50	0.8	0.82	476	17
2015	51	0.77	1.65	476	17
2015	52	0.68	0.76	478	17
2015	53	0.43	0.76	478	17
2016	1	0.91	1.65	476	17
2016	2	0.72	1.88	478	17
2016	3	0.58	1.35	478	17
2016	4	0.52	0.76	477	17
2016	5	0.55	0.59	478	17
2016	6	0.48	0.65	478	17
2016	7	0.52	0.94	478	17
2016	8	0.48	0.88	478	17
2016	9	0.44	1.12	476	17
2016	10	0.5	0.88	478	17
2016	11	0.44	0.47	477	17
2016	12	0.35	1	478	17
2016	13	0.4	0.59	478	17
2016	14	0.33	0.47	478	17
2016	15	0.31	0.59	478	17
2016	16	0.36	0.76	478	17
2016	17	0.47	0.82	477	17
2016	18	0.43	0.35	478	17
2016	19	0.44	0.35	477	17
2016	20	0.53	0.71	478	17
2016	21	0.56	0.65	478	17
2016	22	0.55	0.76	478	17
2016	23	0.58	1.29	477	17
2016	24	0.64	0.41	478	17
2016	25	0.7	0.59	478	17
2016	26	0.64	1.06	478	17
2016	27	0.77	1.41	478	17
2016	28	0.91	1.53	478	17
2016	29	0.79	1.29	478	17
2016	30	1	1.53	477	17
2016	31	0.93	1.59	477	17
2016	32	0.93	1.71	477	17
2016	33	0.88	2.06	478	17
2016	34	0.86	1.29	478	17
2016	35	0.88	1.35	476	17
2016	36	0.85	1.41	477	17
2016	37	0.95	1.29	477	17
2016	38	0.84	1.18	478	17
2016	39	1.18	1.76	478	17
2016	40	1.33	1.65	478	17
2016	41	1.23	1.12	478	17
2016	42	1.61	3	478	17
2016	43	1.46	2.59	478	17
2016	44	1.12	1.65	478	17
2016	45	1.33	1.29	477	17
2016	46	1.32	1.76	477	17
2016	47	0.98	1.12	476	17
2016	48	1.13	1	478	17
2016	49	1.21	2.47	477	17
2016	50	1.01	1.06	478	17
2016	51	0.84	1.24	478	17
2016	52	0.76	1.06	477	17
2017	1	0.67	0.71		
2017	2	0.72	0.94		
2017	3	0.62	1.12		
2017	4	0.51	0.65		
2017	5	0.49	0.47		
2017	6	0.42	0.06		
2017	7	0.38	0.65		
2017	8	0.37	0.76		
2017	9	0.34	0.41		
2017	10	0.34	0.59		
2017	11	0.28	0.35		
2017	12	0.24	0.41		
2017	13	0.26	0.53		
2017	14	0.19	0.13		
2017	15	0.18	0.35		
2017	16	0.23	0.18		
2017	17	0.25	0.24		
2017	18	0.21	0.47		
2017	19	0.25	0.18		
2017	20	0.22	0.47		
2017	21	0.24	0.18		
2017	22	0.25	0.29		
2017	23	0.21	0.18		
2017	24	0.2	0.24		
2017	25	0.24	0.12		
2017	26	0.22	0.35		
2017	27	0.25	0.47		
2017	28	0.25	0.24		
2017	29	0.25	0.41		
2017	30	0.25	0.18		
2017	31	0.25	0.12		
2017	32	0.24	0.41		
2017	33	0.29	0.18		
2017	34	0.26	0.24		
2017	35	0.32	0.24		
2017	36	0.27	0.24		
2017	37	0.32	0.24		
2017	38	0.3	0.12		
2017	39	0.36	0.35		
2017	40	0.32	0.12		
2017	41	0.35	0		
2017	42	0.37	0.35		
2017	43	0.32	0.35		
2017	44	0.39	0.12		
2017	45	0.52	0.35		
2017	46	0.54	0.47		
2017	47	0.34	0.18		
2017	48	0.39	0.12		
2017	49	0.37	0.18		
2017	50	0.37	0.35		
2017	51	0.36	0.12		
2017	52	0.35	0.12		
2018	1	0.2	0.06		
2018	2	0.24	0.24		
2018	3	0.25	0.12		
2018	4	0.2	0.12		
2018	5	0.15	0		
2018	6	0.18	0.18		
2018	7	0.14	0.12		
2018	8	0.13	0.18		
2018	9	0.11	0.12		
2018	10	0.12	0.06		
2018	11	0.2	0.06		
2018	12	0.12	0.06		
2018	13	0.16	0.06		
2018	14				
2018	15				
2018	16				
2018	17				
2018	18				
2018	19				
2018	20				
2018	21				
2018	22				
2018	23				
2018	24				
2018	25				
2018	26				
2018	27				

2018	28		
2018	29		
2018	30		
2018	31		
2018	32		
2018	33		
2018	34		
2018	35		
2018	36		
2018	37		
2018	38		
2018	39		
2018	40		
2018	41		
2018	42		
2018	43		
2018	44		
2018	45		
2018	46		
2018	47		
2018	48		
2018	49		
2018	50		
2018	51		
2018	52		

The latest data are available from the website of the National Institute of Infectious Diseases (https://www.niid.go.jp/niid/ja/idwr.html).
