# Supplementary material for: Genetic characterization of Mycoplasma pneumoniae isolated in Osaka between 2011 and 2017: Decreased detection rate of macrolide-resistance and increase of p1 gene type 2 lineage strains
Source: PLoS One. 2019 Jan 25;14(1):e0209938. doi: 10.1371/journal.pone.0209938 (PMC6347185; doi:10.1371/journal.pone.0209938)
Supplement: S2 Table — (DOCX) [file pone.0209938.s005.docx]

S2 table. Sequences of primers used for sequencing of *p1* operon region.

| 1 | P1-OP-F1 | TACTACTTACAACTCTTTGT |  |
| --- | --- | --- | --- |
| 2 | ORF4-R1 | GTCCCGGCACTCAATCCGTT |  |
| 3 | ORF4-F1 | AGTATTGCGGATTGACCACC |  |
| 4 | ORF4-R2 | TCTTTTGGAACTTCTTATCG |  |
| 5 | 141-200F | GGCTGTTCTTTATAGAAGAG |  |
| 6 | ADH1 | CTGCCTTGTCCAAGTCCACT |  |
| 7 | 141-M3F2 | TCCCCGTCGAGGTGGCGC |  |
| 8 | 141-M4R2 | CGAACTGGAAAGGGCAGT | * |
| 9 | 141-M3R3 | GGGTGCCATCTTGGGTGCCT | * |
| 10 | 141-M3F3 | CAAATGATCCGCCTCGAT | * |
| 11 | 141-M3F4 | AACAAACAAACTGACGGG |  |
| 12 | ADH2 | AACCTTGTCGGGAAGAGCTG |  |
| 13 | ADH3 | CGAGTTTGCTGCTAACGAGT |  |
| 14 | 141-M3F6 | CGCTACGGGGATGGACCA | * |
| 15 | 405-P1-S3-F1 | ATGCGATGGAACAACAAGAG | * |
| 16 | 141-M3F7 | CAGGACGTCGGTAACCTC | * |
| 17 | 141-M3F8 | AGCCCCAATCGCTCAAAA |  |
| 18 | 141-M3R8 | CTGGTCATACACCAACAT |  |
| 19 | 141-M3F9 | ACAGAGCTTAGGTCTCCG | * |
| 20 | ADH4 | CTTGACTGATACCTGTGCGG |  |
| 21 | ORF6-F | GCGCCAAAACGCTTGAAACA |  |
| 22 | 142-M2F1 | ATGAAATCGAAGCTAAAG |  |
| 23 | 141+100R | TGGTCTTGGAGGAGGTAGGT |  |
| 24 | 402-G07-SF1 | CTTTACGACACCGCTCAATG |  |
| 25 | G20S-R8 | CTTTCAGTCCACGAGTTCGT |  |
| 26 | 142-M2R1 | AACTTAATCTTGCGTTCA | * |
| 27 | 142-M2F2 | AAGACCTCCTCGTCGAAC |  |
| 28 | 402-G12-SR1 | GGGTCACGGGTTGTTCGA |  |
| 29 | G20S-F2 | CGGGTGAATGGGGTTGCTCA |  |
| 30 | 402-G12-SR2 | GAACCGTATCCTGAGCAACCC | * |
| 31 | 405-G14S-R2 | CGGAAGTGGTTCACCCATCT | * |
| 32 | 142-M2F3 | CACAGCGAGTGACACCGC | * |
| 33 | 402-G19-SR1 | GGGGTTGTTGGTGAGGTTT | * |
| 34 | 402-G19-SR2 | AGGGGTTCATACACCGGTCA |  |
| 35 | G20S-F3 | AAATATTTAAACACCGCCCA | * |
| 36 | G20S-R7 | GACTGAAACACCACCACCAG | * |
| 37 | G20S-F4 | GACAAGCAGAAGCAGTTTGT |  |
| 38 | 142-M2F4 | AAGGCCCAAGGCCTCACC | * |
| 39 | 142-M2F5 | AGTCGACCTCCGATGATA |  |
| 40 | 142-M2F6 | TACCGGAATTGAATTTAA |  |
| 41 | ORF6-MR | ATCCTTGGTAATCACAGCGG |  |
| 42 | 142-M2F7 | TTCCCTTCCCGGATTTTT |  |
| 43 | ORF6-R | GTGCTTCTTTCTGTTGGGGC | * |
| 44 | P1-OP-R1 | TTGCACTAGGAAGGTAATGT |  |
| 45 | 23e-R1 | AAGAGGTGAAGCCTCGCTAA |  |

* These primers have sequence mismatches depend on variations of *p1* or *orf6* genes.
