# Supplementary material for: Genetic characterization of Mycoplasma pneumoniae isolated in Osaka between 2011 and 2017: Decreased detection rate of macrolide-resistance and increase of p1 gene type 2 lineage strains
Source: PLoS One. 2019 Jan 25;14(1):e0209938. doi: 10.1371/journal.pone.0209938 (PMC6347185; doi:10.1371/journal.pone.0209938)
Supplement: S3 Table — (PDF) [file pone.0209938.s006.pdf]

S3 Table. The list of *M. pneumoniae* isolates collected and analyzed in this study.

No.	Isolate Name	*p1* type	MR mutations	Year	Origin
1	1	1	A2063G	2011	Hospital 9
2	2	1	A2063G	2011	Hospital 9
3	3	1	A2063G	2011	Hospital 9
4	4	1	-	2011	Hospital 9
5	5	1	-	2011	Hospital 9
6	6	1	A2063G	2011	Hospital 9
7	8	1	A2063G	2011	Hospital 9
8	10	1	A2063G	2011	Hospital 9
9	13	1	A2063G	2011	Clinic 1
10	14	2a	-	2011	Clinic 1
11	15	1	A2063G	2011	Clinic 1
12	16	1	A2063G	2011	Clinic 1
13	17	1	A2063G	2011	Clinic 1
14	18	1	A2063G	2011	Clinic 1
15	19	1	A2063G	2011	Clinic 1
16	22	2c	A2063G	2011	Clinic 1
17	23	1	A2063G	2011	Hospital 8
18	24	1	A2063G	2011	Hospital 8
19	25	1	A2063G	2011	Hospital 8
20	27	1	A2063G	2011	Hospital 3
21	28	1	-	2011	Hospital 3
22	29	1	A2063G	2011	Hospital 3
23	32	1	A2063G	2011	Hospital 3
24	33	1	A2063G	2011	Hospital 3
25	34	1	A2063G	2011	Hospital 3
26	35	1	A2063G	2011	Hospital 3
27	36	2a	-	2011	Hospital 3
28	38	2a	-	2011	Hospital 2
29	39	1	A2063G	2011	Hospital 2
30	40	1	A2063G	2011	Hospital 2
31	41	2a	-	2011	Hospital 2
32	42	1	A2063G	2011	Hospital 2
33	43	1	A2063G	2011	Hospital 2
34	44	1	A2063G	2011	Hospital 2
35	45	2c	-	2011	Hospital 2
36	47	1	A2063G	2011	Hospital 2
37	50	2a	-	2011	Clinic 6
38	51	1	A2063G	2011	Clinic 6
39	52	1	-	2011	Clinic 6
40	53	1	-	2011	Clinic 6
41	55	2c	-	2011	Clinic 6
42	56	1	A2063G	2011	Clinic 6
43	M013	1	A2063G	2011	Hospital 2
44	M014	2c	-	2011	Hospital 5
45	M015	1	A2063G	2011	Hospital 5
46	M020	1	A2063G	2011	Hospital 5
47	M021	1	A2063G	2011	Hospital 5
48	M022	1	A2063G	2011	Hospital 5
49	M024	1	A2063G	2011	Hospital 5
50	M025	1	A2063G	2011	Hospital 5

51	M026	1	A2063G	2011	Hospital 5
52	M027	1	A2063G	2011	Hospital 5
53	M029	1	A2063G	2011	Hospital 5
54	M030	1	A2063G	2011	Hospital 5
55	M031	1	A2063G	2011	Hospital 5
56	M032	2b	-	2011	Hospital 5
57	M033	1	A2063G	2011	Hospital 5
58	M034	1	A2063G	2011	Clinic 1
59	M035	1	A2063G	2011	Clinic 6
60	M038	1	A2063G	2011	Hospital 3
61	M039	1	A2063G	2011	Hospital 3
62	M040	1	A2063G	2011	Hospital 9
63	M041	1	A2063G	2011	Hospital 2
64	M059	2a	-	2011	Clinic 1
65	M001	1	A2063G	2012	Hospital 5
66	M002	1	A2063G	2012	Hospital 5
67	M003	1	A2063G	2012	Hospital 5
68	M004	1	A2063G	2012	Hospital 1
69	M005	1	A2063G	2012	Hospital 1
70	M006	1	A2063G	2012	Hospital 5
71	M007	2c	-	2012	Hospital 5
72	M008	1	A2063G	2012	Hospital 6
73	M009	1	-	2012	Hospital 7
74	M023	1	A2063G	2012	Hospital 5
75	M028	1	A2063G	2012	Hospital 5
76	M010	1	A2063T	2013	Hospital 5
77	M011	1	A2063G	2013	Clinic 1
78	M012	2c	-	2013	Clinic 1
79	M016	1	A2063G	2013	Clinic 3
80	M017	1	A2063G	2013	Hospital 2
81	M043	1	A2063G	2013	Hospital 5
82	M044	2c	-	2013	Hospital 5
83	M018	2c	-	2014	Hospital 2
84	M019	1	A2063G	2014	Hospital 2
85	M036	1	A2063G	2014	Hospital 4
86	M037	1	A2063G	2014	Hospital 4
87	M042	1	A2063G	2014	Hospital 4
88	M045	2c	-	2014	Hospital 5
89	M046	1	A2063G	2014	Hospital 5
90	M047	1	A2063G	2014	Hospital 4
91	M048	1	A2063G	2014	Hospital 4
92	M049	1	A2063G	2014	Clinic 3
93	M050	1	A2063G	2014	Clinic 2
94	M051	2c	-	2014	Hospital 4
95	M052	1	A2063G	2014	Hospital 4
96	M053	1	A2063G	2014	Hospital 4
97	M054	2c	-	2014	Hospital 5
98	M055	2c	A2063G	2015	Hospital 4
99	M056	2c	-	2015	Hospital 5
100	M057	1	A2063G	2015	Hospital 5
101	M058	2c	-	2015	Hospital 4
102	M060	2c	A2063G	2015	Hospital 4
103	M061	2c	A2063G	2015	Hospital 4

104	M062	1	A2063G	2015	Hospital 4
105	M063	2	-	2015	Clinic 1
106	M064	2c	-	2015	Clinic 5
107	M065	1	A2063G	2015	Hospital 4
108	M066	2c	-	2015	Clinic 5
109	M067	2c	-	2015	Clinic 1
110	M068	1	A2063G	2015	Hospital 5
111	M069	2c	-	2015	Hospital 5
112	M070	2	-	2015	Clinic 1
113	M071	1	A2063G	2015	Hospital 4
114	M072	2c	-	2015	Hospital 4
115	M073	1	A2063G	2015	Hospital 4
116	M074	1	A2063G	2015	Hospital 4
117	M075	2	-	2015	Hospital 2
118	M076	2	-	2015	Clinic 1
119	M077	2	-	2015	Clinic 1
120	M078	2c	-	2015	Hospital 2
121	M079	1	A2063G	2015	Hospital 4
122	M080	2c	-	2015	Hospital 4
123	M081	2	-	2015	Clinic 1
124	M082	1	A2063G	2015	Hospital 4
125	M083	1	A2063G	2015	Hospital 2
126	M084	1	A2063G	2015	Hospital 4
127	M085	1	A2063G	2015	Hospital 4
128	M086	2	-	2015	Clinic 1
129	M087	2	-	2015	Clinic 1
130	M088	1	A2063G	2015	Hospital 4
131	M089	1	A2063G	2015	Hospital 4
132	M090	1	A2063G	2015	Hospital 2
133	M091	1	A2063G	2015	Hospital 4
134	M092	1	A2063G	2015	Hospital 4
135	M093	2	-	2015	Clinic 1
136	M094	1	A2063G	2015	Hospital 4
137	M095	2c	-	2015	Hospital 4
138	M096	2c	-	2015	Hospital 4
139	M097	2	-	2015	Clinic 1
140	M098	1	A2063G	2015	Hospital 2
141	M099	2c	-	2015	Hospital 4
142	M100	1	A2063G	2015	Hospital 4
143	M101	2c	-	2015	Hospital 2
144	M102	1	A2063G	2015	Hospital 4
145	M103	1	A2063G	2015	Hospital 5
146	M104	1	A2063G	2015	Hospital 2
147	M105	1	A2063G	2015	Hospital 4
148	M106	2	-	2015	Clinic 1
149	M107	2	-	2015	Hospital 5
150	M108	2	-	2015	Hospital 5
151	M109	2	-	2015	Hospital 5
152	M110	2c	-	2015	Hospital 5
153	M111	2c	-	2015	Clinic 4
154	M112	2	-	2015	Hospital 5
155	M113	2	-	2015	Clinic 1
156	M114	2	-	2015	Hospital 5

157	M115	1	A2063G	2015	Clinic 4
158	M116	1	A2063G	2015	Hospital 4
159	M117	1	A2063G	2015	Hospital 4
160	M118	2	-	2015	Clinic 1
161	M119	2c	-	2015	Hospital 2
162	M120	1	-	2015	Hospital 4
163	M121	1	A2063G	2015	Hospital 4
164	M122	1	A2063G	2015	Hospital 5
165	M123	2	-	2015	Clinic 1
166	M124	1	-	2015	Clinic 1
167	M125	1	A2063G	2015	Hospital 4
168	M126	1	A2063G	2015	Hospital 4
169	M127	1	A2063G	2015	Hospital 4
170	M128	1	-	2015	Hospital 5
171	M129	2c	-	2015	Hospital 4
172	M130	2	-	2015	Clinic 1
173	M131	2	-	2015	Clinic 1
174	M132	2	-	2015	Hospital 4
175	M133	2	-	2015	Clinic 1
176	M134	2	-	2015	Clinic 5
177	M135	1	A2063G	2015	Hospital 4
178	M136	2	-	2015	Hospital 5
179	M137	1	A2063G	2015	Clinic 5
180	M138	2	-	2015	Hospital 4
181	M139	2	-	2015	Hospital 5
182	M140	1	A2063G	2015	Hospital 4
183	M141	2c	-	2015	Hospital 4
184	M142	2c	-	2015	Hospital 5
185	M143	2	-	2015	Hospital 5
186	M144	1	A2063G	2015	Hospital 2
187	M145	1	A2063G	2015	Hospital 4
188	M146	2c	-	2015	Hospital 4
189	M147	2	-	2015	Clinic 1
190	M148	2	-	2015	Clinic 1
191	M149	1	A2063G	2015	Hospital 2
192	M150	2c	-	2015	Hospital 2
193	M152	2	-	2015	Hospital 5
194	M153	2	-	2015	Hospital 5
195	M154	1	A2063G	2015	Hospital 5
196	M155	2c	-	2015	Hospital 4
197	M156	2	-	2015	Hospital 5
198	M157	2c	-	2015	Hospital 5
199	M158	1	A2063G	2015	Hospital 4
200	M159	2c	-	2015	Hospital 5
201	M160	1	-	2015	Hospital 4
202	M161	2c	A2063G	2015	Hospital 4
203	M162	1	A2063G	2015	Hospital 5
204	M163	1	A2063G	2015	Hospital 4
205	M164	1	A2063G	2015	Clinic 4
206	M165	1	A2063G	2015	Clinic 1
207	M166	2	-	2015	Clinic 1
208	M167	2	-	2015	Clinic 5
209	M168	2c	-	2015	Clinic 1

210	M169	2	-	2015	Clinic 5
211	M170	2c	-	2015	Hospital 4
212	M171	1	A2063G	2015	Hospital 2
213	M172	1	A2063G	2015	Hospital 4
214	M173	1	A2063G	2015	Hospital 5
215	M174	1	A2063G	2015	Hospital 5
216	M175	1	A2063G	2015	Hospital 5
217	M176	2	-	2015	Clinic 5
218	M177	1	A2063G	2015	Hospital 4
219	M178	2c	-	2015	Clinic 5
220	M179	2c	-	2015	Hospital 4
221	M180	2c	-	2015	Hospital 2
222	M181	1	A2063G	2015	Hospital 4
223	M182	1	A2063G	2015	Hospital 4
224	M183	1	A2063G	2015	Clinic 4
225	M184	2	-	2015	Hospital 5
226	M185	1	A2063G	2015	Hospital 4
227	M186	1	A2063G	2015	Hospital 2
228	M187	1	A2063G	2015	Hospital 2
229	M188	1	A2063G	2015	Clinic 4
230	M189	2	-	2015	Hospital 5
231	M190	2c	-	2015	Clinic 5
232	M191	1	-	2015	Hospital 5
233	M192	2	-	2015	Clinic 1
234	M193	2	-	2015	Clinic 1
235	M194	2	-	2015	Clinic 5
236	M195	2c	-	2015	Clinic 5
237	M196	2	-	2015	Hospital 2
238	M197	1	-	2015	Hospital 5
239	M198	2c	-	2015	Hospital 5
240	M199	1	A2063G	2015	Hospital 5
241	M200	1	A2063G	2015	Hospital 2
242	M202	2	-	2015	Clinic 5
243	M203	2	-	2015	Clinic 1
244	M204	2	-	2015	Clinic 1
245	M205	2c	-	2015	Hospital 4
246	M206	2	-	2015	Clinic 5
247	M207	2c	-	2015	Clinic 1
248	M208	2c	-	2015	Clinic 1
249	M209	1	A2063G	2015	Hospital 5
250	M210	1	-	2015	Hospital 5
251	M215	2c	-	2015	Hospital 5
252	M216	2c	-	2015	Hospital 4
253	M217	1	A2063G	2015	Hospital 5
254	M219	2c	-	2015	Hospital 5
255	M201	1	A2063G	2016	Hospital 2
256	M211	2c	-	2016	Clinic 1
257	M212	2	-	2016	Clinic 1
258	M213	2c	-	2016	Clinic 1
259	M214	2	-	2016	Clinic 1
260	M218	1	A2063G	2016	Hospital 5
261	M220	2	-	2016	Hospital 5
262	M221	1	A2063G	2016	Hospital 5

263	M222	1	-	2016	Hospital 5
264	M223	1	A2063G	2016	Hospital 2
265	M224	2	-	2016	Clinic 1
266	M225	2	-	2016	Hospital 5
267	M226	1	-	2016	Hospital 5
268	M227	2c	-	2016	Hospital 5
269	M228	2c	-	2016	Hospital 5
270	M229	2	-	2016	Hospital 5
271	M230	1	A2063G	2016	Clinic 4
272	M231	1	A2063G	2016	Hospital 5
273	M232	2	-	2016	Hospital 5
274	M233	1	-	2016	Hospital 2
275	M234	1	A2063G	2016	Hospital 2
276	M235	2c	-	2016	Hospital 5
277	M236	2	-	2016	Hospital 5
278	M237	2	-	2016	Hospital 5
279	M238	2	-	2016	Hospital 5
280	M239	2	-	2016	Hospital 5
281	M240	2	-	2016	Hospital 5
282	M241	NT	-	2016	Clinic 1
283	M245	2c	-	2016	Clinic 1
284	M246	2c	-	2016	Clinic 1
285	M247	1	A2063G	2016	Hospital 5
286	M248	2	-	2016	Hospital 5
287	M249	2c	-	2016	Clinic 1
288	M250	2	-	2016	Hospital 5
289	M251	1	A2063G	2016	Hospital 5
290	M252	2c	-	2016	Hospital 5
291	M253	2	A2063G	2016	Clinic 1
292	M254	2	-	2016	Hospital 5
293	M255	1	A2063G	2016	Hospital 5
294	M256	1	A2063G	2016	Hospital 5
295	M257	2	-	2016	Clinic 1
296	M258	2	-	2016	Clinic 1
297	M259	2c	-	2016	Hospital 5
298	M260	2c	-	2016	Clinic 1
299	M261	2	-	2016	Hospital 5
300	M262	2c	-	2016	Hospital 5
301	M263	2c	-	2016	Clinic 1
302	M264	2	-	2016	Clinic 1
303	M265	1	A2063G	2016	Hospital 5
304	M266	2	-	2016	Hospital 5
305	M267	1	A2063G	2016	Hospital 5
306	M268	1	-	2016	Hospital 5
307	M269	2	-	2016	Hospital 5
308	M270	2c	-	2016	Hospital 5
309	M271	1	A2063G	2016	Hospital 5
310	M272	1	A2063G	2016	Clinic 1
311	M273	2	-	2016	Clinic 1
312	M274	2	-	2016	Hospital 5
313	M275	2	-	2016	Hospital 5
314	M276	1	A2063G	2016	Hospital 5
315	M277	1	A2063G	2016	Hospital 5

316	M278	1	A2063G	2016	Clinic 4
317	M279	1	A2063G	2016	Clinic 1
318	M281	2c	-	2016	Hospital 5
319	M282	2f	-	2016	Clinic 4
320	M289	1	-	2016	Hospital 5
321	M290	2	-	2016	Hospital 5
322	M291	1	A2063G	2016	Hospital 5
323	M292	2c	-	2016	Hospital 5
324	M293	2	-	2016	Hospital 5
325	M294	1	A2063G	2016	Hospital 5
326	M295	2	-	2016	Hospital 5
327	M296	1	A2063G	2016	Hospital 5
328	M297	2	-	2016	Clinic 1
329	M298	1	A2063G	2016	Hospital 5
330	M299	2	-	2016	Hospital 5
331	M300	2c	-	2016	Hospital 5
332	M301	2c	-	2016	Hospital 5
333	M302	2c	-	2016	Hospital 5
334	M303	2	-	2016	Hospital 5
335	M305	2	-	2016	Hospital 5
336	M306	2c	-	2016	Hospital 5
337	M307	2	-	2016	Hospital 5
338	M308	2c	-	2016	Hospital 5
339	M309 (K708)	2g	-	2016	Hospital 5
340	M310	1	A2063G	2016	Hospital 5
341	M311	2c	-	2016	Hospital 5
342	M312	2	-	2016	Hospital 5
343	M313	1	A2063G	2016	Hospital 5
344	M314	1	A2063G	2016	Hospital 5
345	M315	2c	-	2016	Hospital 5
346	M316	1	A2063G	2016	Hospital 5
347	M317	2c	-	2016	Hospital 5
348	M318	2	-	2016	Hospital 5
349	M319	1	A2063G	2016	Hospital 5
350	M320	2	-	2016	Hospital 5
351	M321	1	A2063G	2016	Hospital 5
352	M322	1	A2063G	2016	Hospital 5
353	M323	2	-	2016	Hospital 5
354	M324	1	A2063G	2016	Hospital 5
355	M325	1	A2063G	2016	Hospital 5
356	M326	1	A2063G	2016	Hospital 5
357	M327	1	A2063G	2016	Hospital 5
358	M328	2	-	2016	Hospital 5
359	M329	2	-	2016	Hospital 5
360	M330	2	-	2016	Hospital 5
361	M331	2c	-	2016	Hospital 5
362	M332	2	-	2016	Hospital 5
363	M333	1	A2063G	2016	Hospital 5
364	M334	1	A2063G	2016	Hospital 5
365	M335	1	A2063G	2016	Hospital 5
366	M336	1	A2063G	2016	Hospital 5
367	M337	1	A2063G	2016	Hospital 5
368	M338	1	A2063G	2016	Hospital 5

369	M339	1	A2063G	2016	Hospital 5
370	M340	1	A2063G	2016	Hospital 5
371	M341	1	A2063G	2016	Hospital 5
372	M342	1	A2063G	2016	Hospital 5
373	M343	1	A2063G	2016	Hospital 5
374	M344	1	A2063G	2016	Hospital 5
375	M345	1	A2063G	2016	Hospital 5
376	M346	1	A2063G	2016	Hospital 5
377	M347	1	A2063G	2016	Hospital 5
378	M348	1	A2063G	2016	Hospital 5
379	M349	1	A2063G	2016	Hospital 5
380	M350	1	A2063G	2016	Hospital 5
381	M351	1	A2063G	2016	Hospital 5
382	M352	1	A2063G	2016	Hospital 5
383	M353	1	A2063G	2016	Hospital 5
384	M354	2	-	2016	Hospital 5
385	M355	2c	-	2016	Hospital 5
386	M356	1	A2063G	2016	Hospital 5
387	M357	2	-	2016	Hospital 5
388	M358	2	-	2016	Hospital 5
389	M359	1	A2063G	2016	Hospital 5
390	M360	1	A2063G	2016	Hospital 5
391	M361	2	-	2016	Hospital 5
392	M362	1	A2063G	2016	Hospital 5
393	M363	1	A2063G	2016	Hospital 5
394	M364	1	A2063G	2016	Hospital 5
395	M365	2	-	2016	Hospital 5
396	M366	1	A2063G	2016	Hospital 5
397	M367	1	A2063G	2016	Hospital 5
398	M368	2c	-	2016	Hospital 5
399	M369	1	A2063G	2016	Hospital 5
400	M370	1	A2063G	2016	Hospital 5
401	M371	2	-	2016	Hospital 5
402	M372	2	-	2016	Hospital 5
403	M373	1	A2063G	2016	Hospital 5
404	M374	2	-	2016	Hospital 5
405	M375	2	-	2016	Hospital 5
406	M376	2	-	2016	Hospital 5
407	M377	2	-	2016	Hospital 5
408	M378	2	-	2016	Hospital 5
409	M379	2c	-	2016	Hospital 5
410	M380	2	-	2016	Hospital 5
411	M381	2	-	2016	Hospital 5
412	M280	1	A2063G	2017	Clinic 1
413	M283	1	A2063G	2017	Clinic 1
414	M284	2c	-	2017	Clinic 1
415	M285	1	-	2017	Clinic 4
416	M286	1	A2063G	2017	Clinic 1
417	M287	1	A2063G	2017	Clinic 1
418	M288	2c	-	2017	Clinic 1
419	M304	2c	-	2017	Clinic 4
