# Supplementary material for: Genetic characterization of Mycoplasma pneumoniae isolated in Osaka between 2011 and 2017: Decreased detection rate of macrolide-resistance and increase of p1 gene type 2 lineage strains
Source: PLoS One. 2019 Jan 25;14(1):e0209938. doi: 10.1371/journal.pone.0209938 (PMC6347185; doi:10.1371/journal.pone.0209938)

S4 Table. The values used to build the graph of Fig 4.

Annual number of isolates categorized by the MR and *p1* type

Macrolide resistance (MR)	*p1* type	Year						
		2011	2012	2013	2014	2015	2016	2017
MSMP	Type 2 lineage	10	1	2	4	85	85	3
	Type 1	5	1	0	0	7	5	1
MRMP	Type 2 lineage	1	0	0	0	4	1	0
	Type 1	48	9	5	11	61	66	4
	Total	64	11	7	15	157	157 *	8

419

* Three strains M241, M282 (type 2f), and K708 (Type 2g) were included in the Type 2 lineage based on their genetic feature (see text).

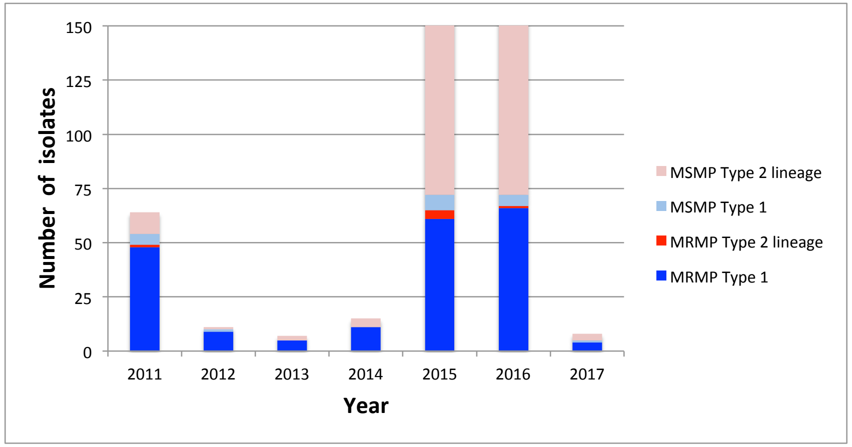

Annual rate of isolates categorized by the MR and *p1* type

Macrolide resistance (MR)	*p1* type	Year						
		2011	2012	2013	2014	2015	2016	2017
MSMP	Type 2 lineage	15.6	9.1	28.6	26.7	54.1	54.1	37.5
	Type 1	7.8	9.1	0	0	4.5	3.2	12.5
MRMP	Type 2 lineage	1.6	0	0	0	2.5	0.6	0
	Type 1	75.0	81.8	71.4	73.3	38.9	42.0	50.0

%

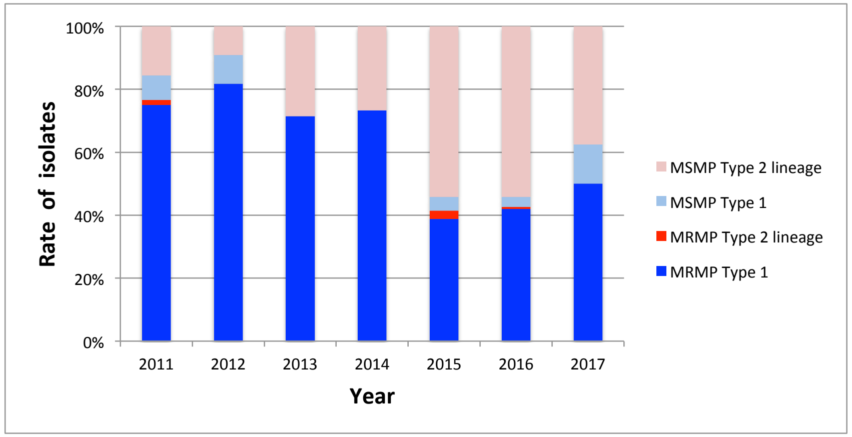

Supplement: S4 Table — (PDF) [file pone.0209938.s007.pdf]
